# Knowledge Discovery and Drug-Repurposing Framework for Pancreatic Ductal Adenocarcinoma: Molecular Networking and Computational Docking

**DOI:** 10.34133/csbj.0067

**Published:** 2026-05-05

**Authors:** Tarik Corbo, Elisabeth Pimpisa Graarud, Mathilde Resell, Abdurahim Kalajdzic, Naris Pojskic, Duan Chen, Björn I. Gustafsson, Chun-Mei Zhao

**Affiliations:** ^1^ Laboratory for Bioinformatics and Biostatistics, University of Sarajevo - Institute for Genetic Engineering and Biotechnology, Sarajevo, Bosnia and Herzegovina.; ^2^ Department of Clinical and Molecular Medicine, Norwegian University of Science and Technology, Trondheim, Norway.; ^3^ Division of Gastroenterology, Department of Medicine, St. Olavs Hospital, Trondheim, Norway.; ^4^ Clinical Academic Group of Cancer Neuroscience, Samarbeidsorganet Helse-Midt Norge, Norway.

## Abstract

Pancreatic ductal adenocarcinoma (PDAC) remains one of the most lethal malignancies, driven by profound molecular heterogeneity and resistance to current therapy. To support systematic target identification, we established a proteomics-anchored knowledge discovery framework integrating cross-model proteomics harmonization, network topology, high-confidence structural modeling, and large-scale in silico docking. From 1,975 proteins consistently detected across murine and human PDAC models, 32 immunohistochemically confirmed candidates were prioritized for structure-based screening against 7,509 clinically characterized compounds. Blind docking, refined pose sampling, ligand-efficiency scoring, and ADME filtering identified EIF2A, STAM, ANXA2, and AHNAK2 as robustly druggable targets. These proteins exhibited high-affinity interactions with zavegepant (a clinically approved CGRP receptor antagonist), omilancor, bemcentinib, conivaptan, and APTO-253. Docking validation (RMSD 1.98 to 2.56 Å) confirmed methodological reliability, and network analyses placed the 4 proteins within modules linked to endosomal/membrane trafficking and invasive phenotypes. Survival analyses in 176 PDAC patients further supported their clinical relevance. Thus, we suggest a systems-level platform for nominating ligandable PDAC targets and clinically actionable compounds. The framework highlights opportunities for rational drug repurposing and motivates future mechanistic studies at the intersection of proteomics and structure-based screening for targets to PDAC.

## Graphical Abstract

**Figure:**
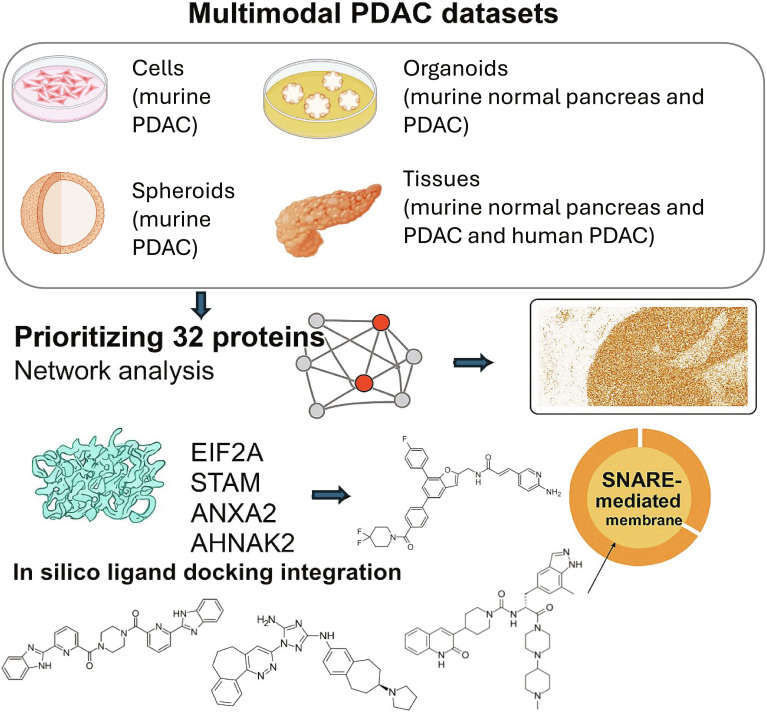

## Introduction

Pancreatic ductal adenocarcinoma (PDAC) accounts for over 90% of pancreatic malignancies and remains among the most lethal cancers, with a 5-year survival rate of approximately 13%. This poor prognosis reflects not only late-stage detection but also profound resistance to conventional therapies, including chemotherapy, radiotherapy, targeted agents, and immunotherapy [1–3]. Although genomic and transcriptomic profiling has revealed recurrent driver mutations such as KRAS, TP53, CDKN2A, and SMAD4, therapeutic targeting of these alterations has yielded limited success due to their intrinsic undruggability and the complexity of downstream signaling networks [4–6]. For instance, C3a/C3aR signaling promoted gemcitabine resistance by sustaining proliferation and migration, underscoring the importance of capturing noncanonical resistance pathways in target discovery efforts [7]. Another layer of PDAC complexity arises from exosome-mediated intercellular communication, which shapes immune evasion and tumor progression. Integration of single-nucleus and exosomal RNA sequencing identified miR-1293 as a clinically relevant biomarker that promotes immunosuppressive phenotypes [8]. These findings highlight the need for computational pipelines that incorporate proteins involved in vesicle trafficking, membrane fusion, and secretion. Additionally, PDAC is driven by profound reprogramming of differentiation and epithelial–mesenchymal transition (EMT) states. Transforming growth factor-β1 (TGF-β1)-induced EMT through Smad4-dependent regulation of Hedgehog–Gli1 signaling is a key axis promoting invasion and stromal remodeling [9]. Effective target prioritization should therefore integrate network-based contextualization of cytoskeletal, trafficking, and signaling proteins. Machine learning (ML)-integrated transcriptomic studies have also identified PDAC prognostic subnetworks [10], and GETdb provides evolutionary/druggability evidence for therapeutic targets [11]. Together, these advances call for computational and structural biotech platforms that can harmonize omics, network inference, structure analyses, and drug–target interaction modeling.

Transcriptomics has been widely applied to the study of many cancer types; however, its application to PDAC remains challenging, particularly when using nonmalignant or normal pancreatic tissue for comparison. This limitation is primarily due to difficulties in obtaining high-quality RNA from pancreatic tissue, which stem from the high levels of ribonuclease and protease digestive enzymes present in pancreatic acinar cells. Of note, this difficulty is particularly considered when using normal pancreatic tissue as a control in studying PDAC. Thus, proteomic investigations might reveal reliable biomarkers, signaling axes, and metabolic regulators that may be amenable to therapeutic modulation [12,13]. Another major challenge persists, i.e., the differences among experimental models that mimic human PDAC. To address these challenges, we have applied an integrative “knowledge discovery in datasets” (KDD) and systems-level modeling approaches that harmonize multimodal proteomic data from models and patients, thereby isolating robust candidate targets that traverse the model–patient divide. Our previous studies have laid the groundwork of this study to further characterize proteomic landscapes across PDAC models and developing computational frameworks for systems-level interpretation [14,15]. In the present work, we further elaborated the methodology, reflecting current PDAC molecular understanding and enabling drug repurposing on the rationale, detailed pipeline, and biological context, situating them within the evolving landscape of PDAC proteomics and therapeutic discovery particularly to address in silico docking and network topology reinforcing their translational potential.

By applying a multimodal proteomics-based KDD workflow across murine and human PDAC models (cells, spheroids, organoids, tissues), we identified proteins consistently present and network-central in both contexts. These candidates were further evaluated through in silico docking against a large ligand library to nominate repurposed small molecules. From an initial set of 1,975 shared proteins, we distilled 32 with confirmable immunohistochemical evidence in PDAC patient tissues. Based on the following definition of druggable target protein that (a) is experimentally verified to be overexpressed in PDAC cells and prognostically relevant, (b) possesses a high-confidence, ligand-able pocket (pLDDT ≥ 70, validated pocket definition), (c) yields at least one repurposed compound with a binding free energy of ≤−9 kcal mol^−1^ and ligand efficiency (LE) of ≥0.30 kcal mol^−1^ per heavy atom, (d) is linked to a disease-relevant network module, and (e) is associated with a clinically characterized small molecule (approved or tested in phase I/II), we found that EIF2A, STAM, ANXA2, and AHNAK2 emerged as the most robustly druggable nodes interacting with repurposed ligands (padnarsertib, bemcentinib, zavegepant).

## Materials and Methods

### Study design

**Fig. 1.**
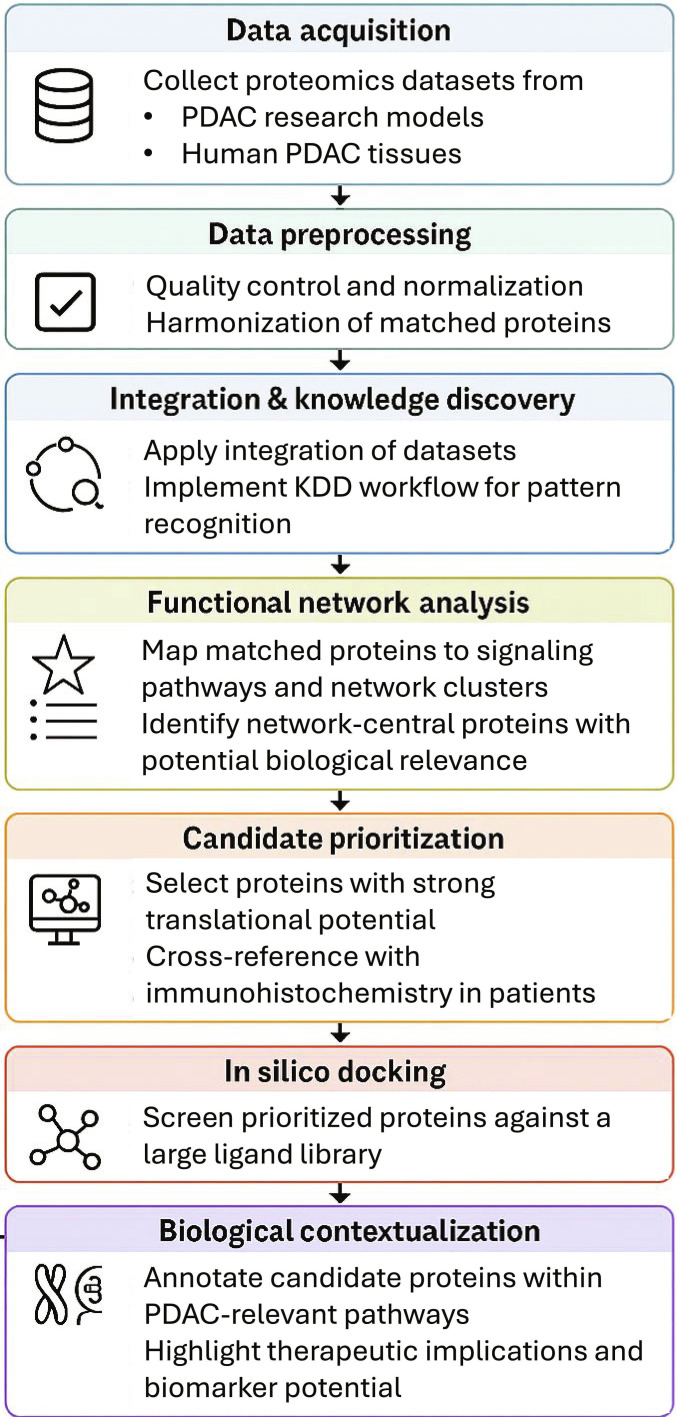
Workflow schematic including 7 steps of data and preprocessing, network and candidates, docking, and biology and translation.

A workflow was established by including multiple steps from data to knowledge discovery using various computational and structure biotechnology (Fig. 1).

### Data collection and preprocessing

Proteomics datasets for PDAC were retrieved from our previous study and are publicly available in established repositories, encompassing both human and murine models of PDAC [14]. Data harmonization and normalization were performed using the KDD pipeline to extract and integrate proteomic profiles across experimental systems, as described previously [15]. Proteomic datasets from murine and human PDAC tissues, spheroids, cell lines, and organoids were harmonized using a shared preprocessing pipeline. Batch correction and missing-value handling were performed using deterministic K-nearest neighbors (KNN) imputation with fixed random seeds. Protein identifiers were unified using UniProt 2025 IDs [16]. Of note, only unambiguous one-to-one orthologs were retained in cross-species integration. Proteins lacking stable orthologs were excluded from shared analyses, ensuring that downstream harmonized datasets reflect biologically comparable molecular entities. Ortholog mapping was qualitative, i.e., used to identify shared protein identities, not to scale expression values across species. As reported previously, the integration of murine and human datasets was qualitative, based on presence/absence across models, not quantitative cross-species expression scaling. The goal was to identify proteins consistently detected in both species, thereby ensuring cross-model robustness [15]. No cross-species intensity normalization was performed, as such quantification requires different experimental designs and was beyond the scope of this study.

The proteomic datasets were normalized using the same KDD harmonization pipeline as described in our previous study [15], employing deterministic KNN imputation with fixed random seeds, global centering and scaling for intra-species comparability, and surrogate variable analysis (SVA)-style batch adjustment within species. Of note, cross-species integration did not involve rescaling intensities between mouse and human; instead, harmonization operated at the feature-selection level (shared orthologs + cross-model consistency).

### Knowledge discovery and protein prioritization

#### Network and pathway analysis

A total of 1,975 unique proteins were previously identified following data integration and feature selection in the KDD workflow [15]. These proteins were subsequently characterized according to degree centrality and topological coefficient to quantify network connectivity and clustering properties within PDAC-associated signaling pathways. Proteins exhibiting high connectivity and centrality scores were considered potential key regulatory nodes and prioritized for further validation. Protein–protein interaction (PPI) network analysis was performed using STRING (v12.0) [17] and visualized in Cytoscape (v3.10.4) [18] to contextualize the docking-selected proteins within the broader PDAC proteomic landscape. All topological metrics (degree, clustering coefficient, topological coefficient) were also calculated within the same 1,973-node STRING-derived PDAC background network, ensuring that metrics were inherently normalized by being computed on an identical node-edge universe and no cross-network comparison was made that could inflate or distort centrality interpretations. Of note, in systems biology and proteomics-anchored workflows, centrality is commonly used as a conservative heuristic for identifying proteins occupying stable, recurrent positions in disease-specific interaction modules, although not as evidence of druggability. In the present study, the centrality helps anchor the prioritization in stable PDAC-enriched regions of the interactome, avoiding false positives that arise from model-specific protein fluctuations.

Network topology was assessed using graph-based parameters including clustering coefficient, neighborhood connectivity, and topological coefficient. Module detection was performed using the Markov cluster algorithm (MCL) [19] to identify densely connected regions of the interactome as well as possible functional modules.

#### PPI network construction, analysis, and clustering

PPI analysis was performed to contextualize the docking-selected proteins within the broader PDAC proteomic landscape. The 1,975 proteins were used as input for network construction. Interaction data were retrieved in Cytoscape using stringApp (v2.2.0) [20] connected to STRING, restricted to *Homo sapiens*. Comparison of the original input list with the STRING/Cytoscape node list showed that most discrepancies reflected identifier harmonization and preferred gene symbol updates rather than exclusion of proteins from the analysis. The final STRING-derived network contained 1,973 nodes, and all 32 docking-selected proteins were represented in this network (Table S1).

For construction and visualization of the global PPI network, a STRING interaction confidence threshold of 0.4 was applied. The resulting network was analyzed in Cytoscape using NetworkAnalyzer [21] to calculate topological parameters including degree, clustering coefficient, betweenness centrality, closeness centrality, and topological coefficient. Network visualization was performed using the Prefuse Force Directed [22] layout to emphasize densely connected regions while preserving global network structure. The full STRING-derived network comprised 1,973 nodes and 74,582 edges. The 32 docking-selected proteins were then mapped onto the full PPI network and visually highlighted to assess their spatial distribution within the PDAC interactome. To characterize their immediate interaction environment, a first-neighbor filtered network containing the docking-selected proteins and their directly connected partners was extracted from the full network. This filtered network comprised 857 nodes and 26,155 edges and was used for focused visualization and downstream module-level analysis (Table S2).

For clustering, the filtered network was subjected to MCL analysis using clusterMaker2 (v2.3.4) [23] together with stringApp. STRING combined scores were used as edge weights, and clustering was performed using an interaction score threshold of 0.7. The MCL inflation parameter was set to 2.5 to achieve moderate cluster granularity. The array source was set to stringdb::score, edges were treated as undirected, and loop adjustment was enabled prior to clustering. Weak edge weights were pruned at 1 × 10^−15^, clustering was iterated 20 times with a maximum residual value of 0.001, and the “stop if residual increases” option was enabled to ensure convergence stability. The clustered network was then used for module-level visualization of docking-selected proteins and their first-neighbor interaction neighborhoods. Proteins were assigned to clusters, followed by the generation of module-level summaries (Table S3).

### Selection according to immunohistochemistry

The Human Protein Atlas (HPA) database, which is created by available antibody-based immunohistochemistry data [24], was used to confirm protein expression in PDAC tissues (https://www.proteinatlas.org/). Proteins demonstrating consistent cytoplasmic, nuclear, or membrane staining in malignant ductal cells were retained as possible druggable targets. Accordingly, 32 proteins met the inclusion criteria and were selected for further in silico analyses.

### 3D structure selection

The 3-dimensional (3D) structures of the target proteins were retrieved in Protein Data Bank (PDB) format from the Research Collaboratory for Structural Bioinformatics (RCSB) PDB and AlphaFold Protein Structure Database [16,25,26]. The dataset included ACADSB (PDB ID: 2JIF), ACTR1A (AlphaFold ID: AF-P61163-F1-v6), AHNAK2 (PDB ID: 4CN0), ANXA2 (PDB ID: 2HYU), ANXA3 (PDB ID: 1AII), ATP6V1F (PDB ID: 6WM2), EIF2A (PDB ID: 8DYS), GSN (PDB ID: 3FFN), HPCAL1 (AlphaFold ID: AF-P37235-F1-v6), MVB12A (PDB ID: 6VME), MYH14 (PDB ID: 5JLH), MYO1C (PDB ID: 4BYF), OTUB1 (PDB ID: 2ZFY), PACSIN2 (PDB ID: 3ABH), PCCA (PDB ID: 7YBU), PCK2 (AlphaFold ID: AF-Q16822-F1-v6), PDHB (PDB ID: 3EXE), PLP2 (AlphaFold ID: AF-Q04941-F1-v6), POLR2H (PDB ID: 7OB9), SCO1 (PDB ID: 2GGT), SCPEP1 (AlphaFold ID: AF-Q9HB40-F1-v6), SERPINB6 (AlphaFold ID: AF-P35237-F1-v6), SFXN2 (AlphaFold ID: AF-Q96NB2-F1-v6), SH3BGRL3 (AlphaFold ID: AF-Q9H299-F1-v6), SNAP23 (AlphaFold ID: AF-O00161-F1-v6), SPTAN1 (PDB ID: 3F31), STAM (AlphaFold ID: AF-Q92783-2-F1-v6), SURF1 (AlphaFold ID: AF-Q15526-F1-v6), TIMM50 (PDB ID: 4QQF), VIL1 (AlphaFold ID: AF-P09327-F1-v6), VTI1B (PDB ID: 2V8S), and YWHAQ (PDB ID: 6BD2). Nonprotein components, including cocrystallized ligands, solvent molecules, and nonrelevant chains, were removed prior to analysis. Structural quality was assessed using crystallographic resolution for PDB structures and predicted local distance difference test (pLDDT) scores for AlphaFold models. AlphaFold-predicted structures were retained in their full-length form without removal of low-confidence regions to preserve structural completeness and avoid bias toward predefined binding sites. Detailed structural confidence metrics, including global (average pLDDT) and binding site-specific pLDDT values, are provided in Table S4.

### Protein structure preparation

Protein preparation was conducted in AutoDock Tools 1.5.6 [27], including removal of water molecules, addition of polar hydrogens, and assignment of Kollman charges. The processed structures were converted to PDBQT format. No structural relaxation or energy minimization was applied prior to docking, and all simulations were performed on full-length protein models. Docking gridboxes were defined individually for each receptor to encompass the entire protein surface, enabling unbiased identification of potential ligand-binding sites. Box dimensions (Å) and centers (*x*, *y*, *z*) were as follows: ACADSB (58 × 58 × 78 Å; center: 91.443, 98.611, 5.426), ACTR1A (64 × 74 × 70 Å; center: −2.484, 0.330, −0.555), AHNAK2 (50 × 54 × 68 Å; center: 26.096, 6.962, −3.512), ANXA2 (65 × 52 × 68 Å; center: 71.499, 9.683, 46.183), ANXA3 (50 × 52 × 70 Å; center: 12.222, −2.229, 1.241), ATP6V1F (40 × 40 × 38 Å; center: 204.891, 191.726, 189.793), EIF2A (58 × 58 × 58 Å; center: −13.672, −8.016, 18.151), GSN (84 × 66 × 88 Å; center: 16.143, 47.125, −22.861), HPCAL1 (60 × 40 × 54 Å; center: −9.309, 8.554, 0.536), MVB12A (30 × 18 × 24 Å; center: 67.281, −33.203, −3.918), MYH14 (82 × 92 × 80 Å; center: 61.697, 9.098, −25.490), MYO1C (70 × 72 × 98 Å; center: −3.301, 6.734, 124.499), OTUB1 (50 × 52 × 38 Å; center: −9.673, −23.064, 11.894), PACSIN2 (72 × 90 × 126 Å; center: −5.863, 7.952, 25.462), PCCA (88 × 78 × 82 Å; center: 237.567, 190.085, 242.678), PCK2 (86 × 58 × 70 Å; center: −12.539, −0.324, −1.776), PDHB (60 × 58 × 50 Å; center: 25.813, −16.966, 64.814), PLP2 (70 × 44 × 60 Å; center: −11.917, −6.517, 5.222), POLR2H (38 × 48 × 44 Å; center: 144.647, 80.772, 161.961), SCO1 (42 × 46 × 38 Å; center: −16.161, −1.362, 27.204), SCPEP1 (58 × 60 × 58 Å; center: −0.175, −3.422, −3.036), SERPINB6 (60 × 52 × 72 Å; center: 0.362, −1.712, −6.814), SFXN2 (70 × 64 × 74 Å; center: −4.454, 1.747, −2.894), SH3BGRL3 (38 × 32 × 42 Å; center: −2.636, −2.411, 2.303), SNAP23 (86 × 60 × 82 Å; center: −0.249, −9.256, 1.012), SPTAN1 (102 × 56 × 56 Å; center: 16.552, 30.444, 26.053), STAM (68 × 68 × 86 Å; center: −16.556, 5.047, 6.614), SURF1 (70 × 52 × 122 Å; center: −1.000, 2.878, −34.240), TIMM50 (40 × 54 × 44 Å; center: −23.151, −10.951, −15.037), VIL1 (86 × 76 × 92 Å; center: 0.991, 3.436, 9.223), VTI1B (48 × 48 × 26 Å; center: 4.959, 1.459, −10.679), YWHAQ (44 × 50 × 64 Å; center: −28.613, 1.799, −16.013). For all docking runs, grid spacing was fixed at 1.0 Å.

### Compound selection and preparation

A library of 7,509 compounds was compiled from ClinicalTrials.gov and the EU Clinical Trials Register and retrieved from PubChem, with gemcitabine included as a positive control to benchmark docking performance [26]. In accordance with studies [28,29], compounds were downloaded in structure data file (SDF) format and converted to PDBQT using OpenBabel 3.1.1. Ligand preparation was performed using OpenBabel 3.1.1 with default settings, which generate neutral 3D conformations with assigned partial charges and explicit hydrogens. Given the large-scale nature of the screening (7,509 compounds) and the multi-target scope of the study, a standardized ligand preparation protocol was applied across all compounds to ensure consistency and comparability of docking results. While pH-dependent protonation and tautomer enumeration can improve accuracy for individual ligand–target systems, such approaches are computationally intensive and are more commonly applied in focused docking or lead optimization studies. Of note, the generated structures correspond to neutral forms as produced by default OpenBabel settings, and no explicit pH-dependent protonation or tautomer enumeration was performed. This standardized approach was applied consistently across all compounds to enable comparative analysis in large-scale virtual screening [30].

### Structural modeling and docking pipeline

In silico docking simulations were performed using AutoDock Vina 1.1.2, applying a blind docking strategy. An initial virtual screening was conducted with an exhaustiveness value of 8 for the full library of 7,509 compounds. Subsequently, the top 50 ligands per target, selected based on docking score, were re-docked using an increased exhaustiveness value of 32 to improve conformational sampling and ensure more reliable binding pose prediction within the large search space. Following docking, the resulting protein–ligand complexes were visualized and analyzed using PyMOL 3.1 and BIOVIA Discovery Studio Visualizer [31,32].

### Docking protocol validation

The docking protocol was validated using a redocking strategy to assess its ability to reproduce experimentally determined ligand-binding poses. Due to the absence of cocrystallized drug-like ligands in the selected target structures, validation was performed using both an external reference complex and a representative structure from the dataset analyzed [33].

Redocking was performed on the crystal structure with PDB ID: 3HS4, containing the cocrystallized ligand acetazolamide, and on PDB ID: 4BYF, which is part of the studied dataset and contains adenosine diphosphate (ADP) orthovanadate as a bound ligand. For each system, the native ligand was extracted, prepared using the same workflow applied to the screened ligands, and re-docked into the corresponding binding site using identical grid box dimensions and docking parameters as in the main study. The predicted poses were aligned with the experimental conformations using PyMOL, and the root mean square deviation (RMSD) of heavy atoms was calculated [30,34].

### LE and ADME analysis

Following the initial docking screen, the top-scoring ligands were further prioritized using ligand efficiency (LE) and absorption, distribution, metabolism, and excretion (ADME) based drug-likeness criteria [31]. LE was used to normalize binding affinity to molecular size, allowing comparison of compounds with different heavy atom counts. In parallel, pharmacokinetic and developability properties were evaluated using SwissADME-derived descriptors, including molecular weight, consensus LogP, topological polar surface area (TPSA), hydrogen-bond donors and acceptors, rotatable bonds, gastrointestinal (GI) absorption, P-glycoprotein (P-gp) substrate status, Lipinski violations, and pan-assay interference compounds (PAINS) alerts. Compounds were prioritized when they combined favorable docking affinity with higher LE and acceptable ADME behavior, particularly high GI absorption, low-to-moderate TPSA, absence of severe P-gp efflux liability, and minimal rule-of-5 violations [32].

### Druggability criteria

A protein is considered druggable if it satisfies all of the following 5 criteria: (a) it is experimentally verified to be overexpressed in PDAC cells and prognostically relevant, (b) it possesses a high-confidence, ligand-able pocket (pLDDT ≥ 70, validated pocket definition), (c) it yields at least one repurposed compound with a binding free energy of ≤−9 kcal mol^−1^ and LE of ≥0.30 kcal mol^−1^ per heavy atom, (d) it is linked to a disease-relevant network module, and (e) it is associated with a clinically characterized small molecule (approved or phase I/II).

### Survival analysis

Clinical prognostic importance was evaluated using RNA expression and survival data of 176 PDAC patients (median follow-up 1.27 years) obtained from the HPA [24]. Patients were stratified into high- and low-expression groups based on median expression levels. Kaplan–Meier survival curves were accessed directly from HPA, and log-rank tests were used to assess statistical significance for each of the 32 candidate genes. Proteins with *P* < 0.001 were considered prognostic biomarkers. Of note, these analyses were unadjusted for clinical covariates and should therefore be interpreted cautiously.

## Results and Discussion

### Robust target prioritization

This study applied a proteomics-anchored knowledge discovery pipeline combined with large-scale structure-based virtual screening to nominate druggable proteins and repurposed small molecules for PDAC. By harmonizing multimodal proteomic datasets from the multiple models and filtering for proteins with confirmatory immunohistochemistry in patient tissue, we generated a list of 32 candidate proteins and then interrogated their structural ligand ability against a curated library of 7,509 clinically relevant compounds. Overall, the in silico screen produced a spectrum of binding energies (roughly −8 to −11.3 kcal/mol) and revealed recurring interaction motifs (hydrogen bonds, halogen contacts, π-stacking, and hydrophobic packing) concentrated in proteins functionally linked to translation control, membrane trafficking, and endosomal/exocytic machinery. These findings both validate the KDD strategy as a way to prioritize targets that are model-agnostic and point to specific, mechanistically plausible protein–drug pairs for experimental follow-up.

### The protein expression in PDAC tissue

**Fig. 2.**
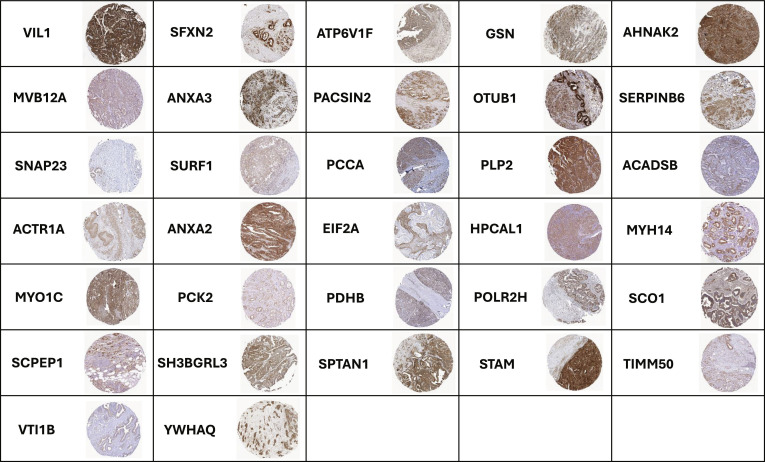
Representative immunohistochemistry using tissue microarrays with available antibodies to 32 of 1,975 proteins (courtesy of HPA). Note: Expression localized to cytoplasm (EIF2A, AHNAK2), nucleus (STAM), and plasma membrane (ANXA2).

HPA immunohistochemistry showed the expression of the panel of 32 proteins in human PDAC tumor tissues (Fig. 2). Only proteins showing consistent cytoplasmic, nuclear, or membrane staining in malignant ductal cells were retained, ensuring tissue-level relevance rather than model-restricted expression.

### Single-cell and subcellular context

**Fig. 3.**
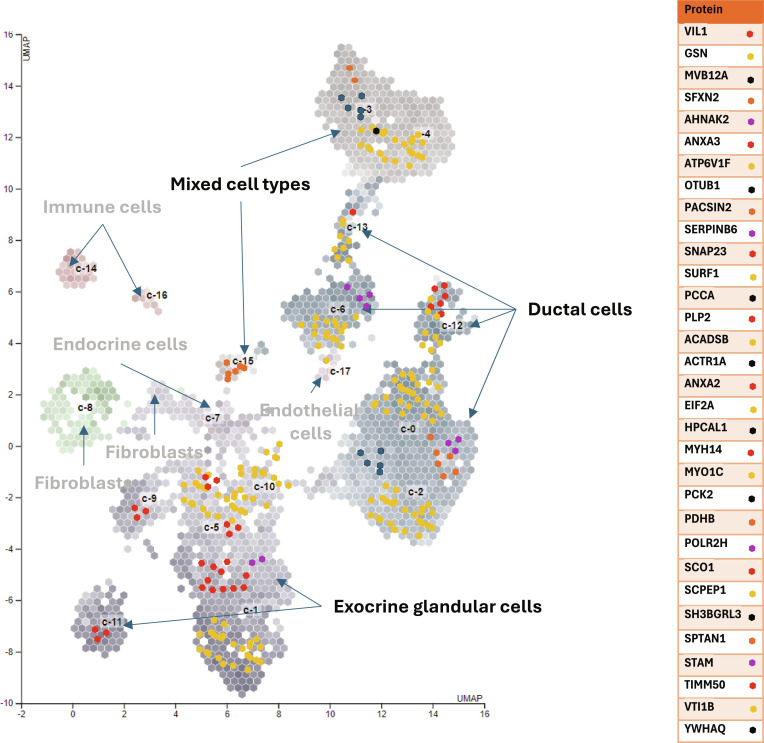
Uniform Manifold Approximation and Projection (UMAP) reference of 32 proteins in the single-cell atlas, with immune, endocrine, endothelial, and fibroblast cells excluded by systems modeling. Colored hexagons show protein locations (not expression levels). EIF2A, STAMBP, ANXA2, and AHNAK2 were expressed in pancreatic ductal cells (c-6, c-12, c-13), localized to cytoplasm (EIF2A, AHNAK2), nucleus (STAM), and plasma membrane (ANXA2) based on the single-cell atlas [35] and protein–protein interactions (IPAs; data not shown).

Integration with single-cell atlases [35] localized the 4 lead candidates to pancreatic ductal cell clusters (c-6, c-12, c-13) (Fig. 3). For example, subcellular annotations [36] indicated EIF2A and AHNAK2 as predominantly cytoplasmic, STAM as nuclear, and ANXA2 as enriched at the plasma membrane, a distribution consistent with their putative roles in translational control (EIF2A), scaffolding/signaling (AHNAK2), endosomal sorting/deubiquitylation (STAM), and membrane dynamics/vesicle trafficking (ANXA2).

### Network topology and target prioritization

#### Docking-selected proteins in a global overview of the PDAC proteomic landscape

To place the docking-selected proteins within a systems-level PDAC context, a STRING-derived PPI network was constructed from the common PDAC protein set. Although the biological input dataset comprised 1,975 proteins, the final STRING/Cytoscape network used for visualization and analysis contained 1,973 nodes after identifier harmonization, and the 32 docking-selected proteins were represented in this network. The full network comprised 74,582 edges, indicating extensive connectivity across the PDAC interactome (Table S2).

**Fig. 4.**
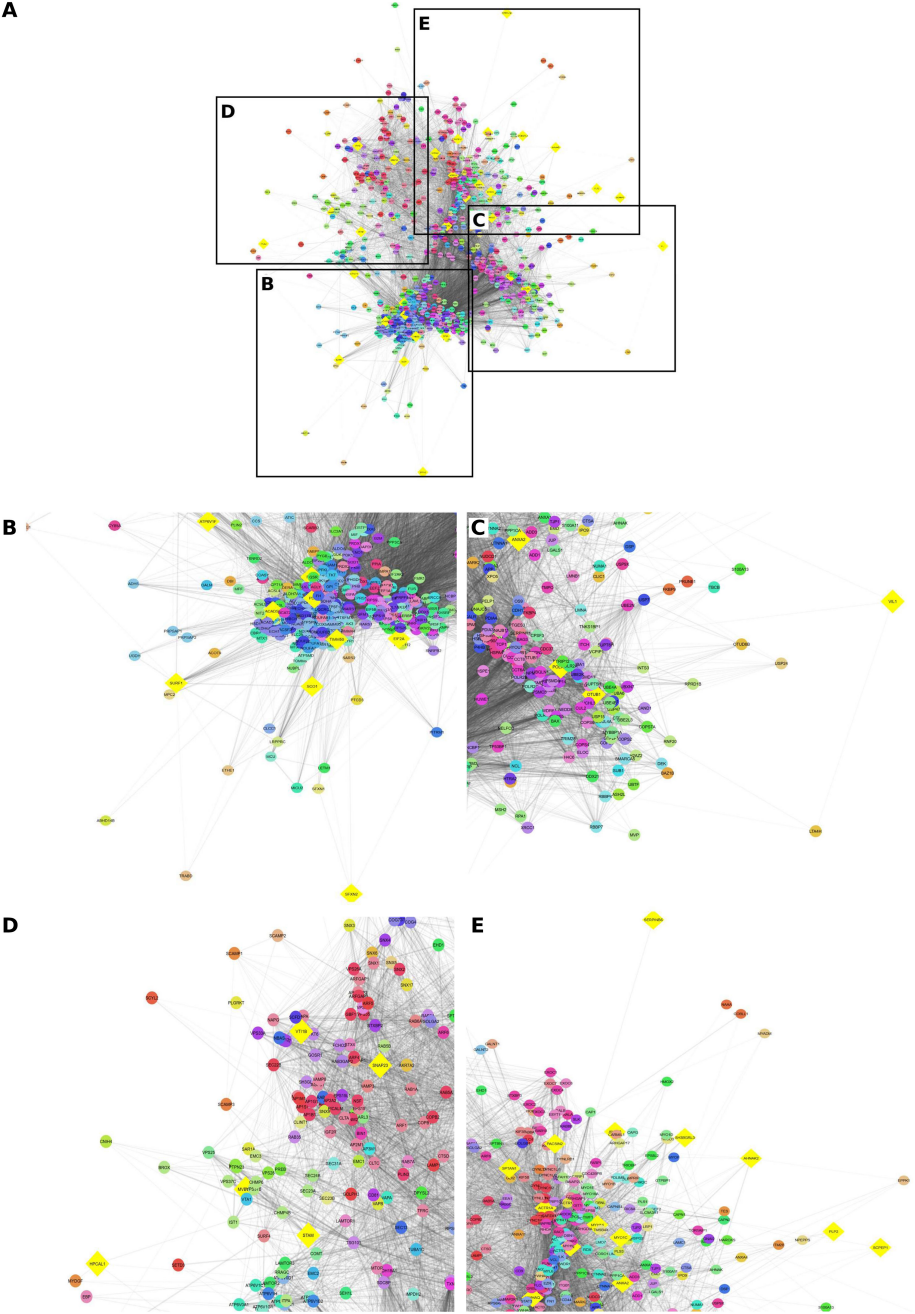
Distribution of docking-selected proteins within the global PDAC protein–protein interaction network. (A) Overview of the STRING-derived PDAC interactome constructed from the common PDAC protein set after identifier harmonization in Cytoscape. The final network contained 1,973 nodes and 74,582 edges. Yellow diamond nodes indicate docking-selected proteins. Black boxes mark the regions enlarged in (B) to (E). (B to E) Zoomed views of representative regions from the global network showing local neighborhoods containing docking-selected proteins. These enlarged panels were included to improve node-level readability and illustrate that docking-selected proteins are distributed across multiple densely connected as well as more peripheral regions of the PDAC interactome. Network summary statistics are provided (Table S2).

The 32 docking-selected proteins were distributed across the global interactome rather than forming one isolated candidate-specific region, indicating that the candidate set is topologically heterogeneous and embedded across multiple parts of the PDAC interaction landscape (Fig. 4).

To further characterize the immediate interaction environment of the docking-selected proteins, a first-neighbor filtered subnetwork containing the 32 docking-selected proteins and their directly connected partners was extracted from the full network. This filtered network comprised 857 nodes and 26,155 edges (Table S2). Retention of a large and densely connected interaction neighborhood after first-neighbor filtering indicates that the docking-selected proteins are structurally integrated within interaction-rich regions of the PDAC proteome. Together, these findings provide network-level context for the candidate set and support subsequent module-based analysis without implying functional validation or therapeutic tractability.

#### MCL clustering reveals distribution of docking-selected proteins across multiple network modules

To resolve the modular organization of the docking protein interaction landscape, the first-neighbor filtered network was clustered using the MCL. The clustered network retained 857 nodes and contained 9,270 edges, indicating that module-level visualization was performed on the same focused interaction set used for first-neighbor analysis (Table S2). Cluster assignments for the docking-selected proteins are summarized, and module-level summaries including module sizes and figure panel assignments are provided in Table S3.

**Fig. 5.**
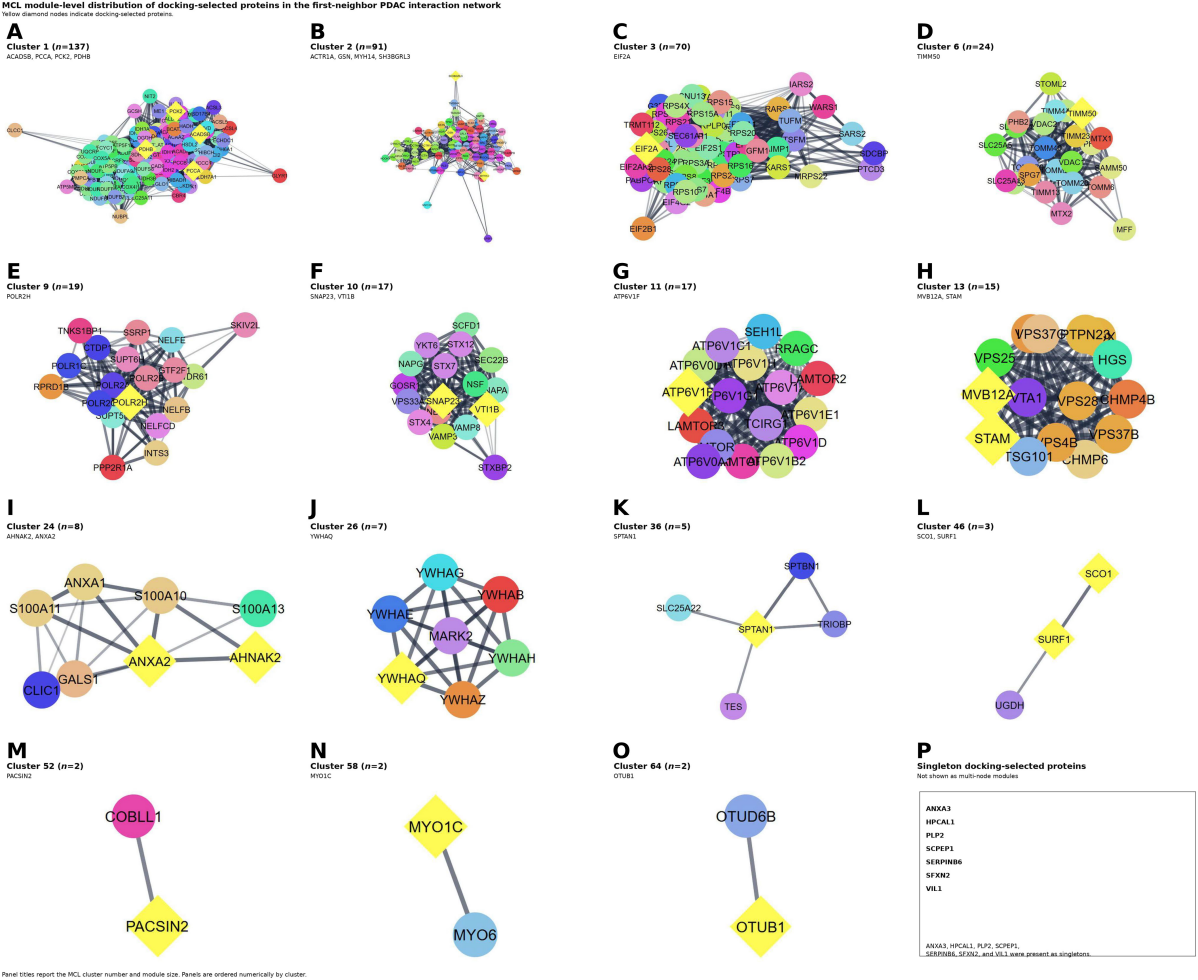
MCL module-level distribution of docking-selected proteins in the first-neighbor PDAC interaction network. (A to O) MCL clusters from the first-neighbor filtered network containing one or more docking-selected proteins and their directly connected interaction partners. Larger and more densely connected modules are shown alongside smaller clusters to permit direct inspection of candidate localization across the clustered network. Yellow diamond nodes indicate docking-selected proteins. (P) Docking-selected proteins present as singletons and therefore not shown as multi-node modules. The clustered network was derived from the 857-node first-neighbor network and shows that the docking-selected proteins are distributed across multiple interaction modules of varying size and density rather than being concentrated within a single cluster. Cluster membership, module size, and candidate-to-module mapping are summarized in Table S3.

The MCL analysis showed that the docking-selected proteins were distributed across several clusters in different sizes rather than concentrated within a single module (Fig. 5).

Of note, this panel layout was adopted to address the readability limitations of the dense network display and to allow direct verification of module structure and candidate placement. Several docking-selected proteins localized within larger and more densely connected clusters, whereas others were present in small, paired modules or as isolated singleton candidates. Accordingly, the candidate set was not confined to one dominant signaling axis but instead spanned multiple topological neighborhoods of the PDAC interactome.

Overall, the MCL clustering analysis complements the global network overview by showing that the docking-selected proteins are embedded in multiple interaction modules of varying size and density. These results therefore provide systems-level context for candidate prioritization and support a network-informed, hypothesis-generating interpretation of the selected proteins.

This interpretation was reinforced by the first-neighbor filtered network, which remained large and densely connected after restricting the analysis to the docking-selected proteins and their direct interactors. Importantly, this connectivity should be interpreted within the scope of the STRING framework. In fact, STRING integrates both physical interactions and broader functional associations derived from multiple evidence channels, including experiments, curated databases, coexpression, conserved genomic context, and text mining [17]. Accordingly, dense network embedding supports biological relatedness and contextual integration within the PDAC proteome, but does not in itself establish direct physical interaction, causal importance, or therapeutic tractability. The present findings should therefore be understood as providing systems-level context for the candidate set rather than functional validation.

The clustering analysis further showed that the docking-selected proteins were distributed across multiple modules of varying size and density rather than accumulating within a single dominant cluster. This modular distribution is concordant with prior protein-level studies in PDAC showing that PDAC biology can be resolved into several biologically distinct networked programs. This is in line with earlier studies that identified 12 coexpressed protein modules associated with processes including metabolism, immune-related signaling, coagulation, and epithelial–mesenchymal transition and with protein network modules linked to different clinical outcome groups in PDAC [16,37,38]. Of note, these earlier studies were based on coexpression rather than STRING-derived PPI clustering.

Based on the present study, we suggest that the distribution of docking-selected proteins across several topological neighborhoods may be considered biologically informative. Candidates located within larger and denser modules may be associated with broader and more interconnected cellular programs, whereas those appearing in smaller clusters or peripheral regions may reflect more specialized or context-dependent aspects of PDAC biology. However, such interpretation remains inferential and should be treated cautiously. Network position and module membership can inform prioritization, but they do not by themselves demonstrate mechanistic relevance or translational utility.

Methodologically, the use of MCL provided a suitable framework for resolving the first-neighbor interaction network into more interpretable graph partitions, as the algorithm is based on a Markov clustering process that alternates expansion and inflation to expose cluster structure in graphs [19], and was adopted early in biological sequence clustering applications such as TRIBE-MCL [39]. In the present study, the value of this approach lies not in the clustering procedure alone, but in its ability to reveal that the docking-selected proteins are embedded across multiple connected regions of the PDAC interactome. Taken together, the global and module-level analyses support a network-informed interpretation of the candidate set and provide a structured basis for subsequent pathway-level analysis and experimental follow-up while remaining clearly distinct from direct functional or therapeutic validation.

### Docking validation

To evaluate the reliability of the docking protocol, redocking experiments were performed using both an external reference complex and a representative structure from the analyzed dataset. For PDB ID 3HS4, the cocrystallized ligand (acetazolamide) was reproduced with an RMSD of 1.98 Å relative to its crystallographic conformation. Additionally, redocking of ADP orthovanadate into the MYO1C structure (PDB ID 4BYF), which was included in the docking study, yielded an RMSD of 2.56 Å.

The obtained RMSD values indicate accurate reproduction of experimental binding poses, with the external reference complex achieving near-experimental accuracy (≤2.0 Å) and the dataset-derived structure remaining within the generally accepted threshold of ≤3.0 Å for reliable docking predictions.

### In silico docking

A computational pipeline was performed using AlphaFold structures as provided, but restricted docking to their high-confidence regions. This approach is consistent with numerous large-scale docking screen workflows using the publicly available structures. Of note, energy minimization, molecular dynamics relaxation, homology-based editing, and disorder truncation were not performed, as structural refinement was outside the scope of the present study. The structural quality of all protein models used for docking was confirmed (Table S5). Experimentally determined structures exhibited high crystallographic resolution (typically ≤2.5 Å), while AlphaFold-predicted models showed high global confidence, with binding site pLDDT ≥ 70, supporting reliable local structural accuracy. These findings validate the suitability of the selected protein models for docking simulations.

In silico molecular docking was performed to evaluate the binding potential of selected compounds across all 32 candidate proteins identified through the KDD workflow. The simulations revealed diverse ligand–protein interaction patterns, including hydrogen bonds, hydrophobic contacts, halogen interactions, and π-stacking interactions, reflecting variability in binding site architecture and ligand physicochemical properties (Table S5).

Among the 32 proteins, EIF2A, ANXA2, AHNAK2, and STAM exhibited the most favorable binding profiles, characterized by consistently high-affinity interactions and complex interaction networks with clinically approved or investigational compounds. These interactions frequently involved residues located within functionally relevant domains associated with translational regulation, membrane trafficking, and signal transduction, supporting their potential as druggable targets in PDAC. In contrast, the positive control, gemcitabine, demonstrated limited interaction profiles across the evaluated targets, primarily forming isolated hydrogen bonds with minimal contribution from hydrophobic or π-mediated interactions. This reduced interaction complexity further highlights the improved binding complementarity and target engagement observed for the top-ranked repurposed compounds.

For EIF2A, ANXA2, AHNAK2, and STAM, the binding pockets lie within consistently ordered, stable domains known from the literature (e.g., ANXA2 core annexin domain, STAM VHS/SH3 domains, EIF2A N-terminal structured region, and AHNAK2 WD-repeat/structured regions). AlphaFold’s confidence maps strongly match known structured domains for these proteins. Docking into low-confidence regions was avoided through pocket-focused grid placement. Of note, the docking results served as the foundation for subsequent ligand prioritization using LE and ADME-related criteria and should be used only as hypothesis-generating evidence, not as definitive structural predictions.

#### Integrated ligand prioritization (LE and ADME)

Ligand prioritization was performed using an integrated framework that simultaneously considered docking affinity, LE, and ADME-related drug-likeness properties. This approach enabled the identification of compounds that combine strong binding potential with efficient molecular size utilization and favorable pharmacokinetic characteristics.

Within this framework, conivaptan emerged as the most suitable candidate for AHNAK2, demonstrating a balanced profile across all evaluated parameters, including strong binding affinity, satisfactory LE, and a favorable ADME profile characterized by high GI absorption, absence of P-gp substrate behavior, and no Lipinski rule violations.

For ANXA2, 3 compounds fulfilled the combined selection criteria: omilancor, bemcentinib, and SYHA1813. Omilancor exhibited the strongest binding affinity within this target, supporting its role as the primary candidate. SYHA1813 demonstrated the highest LE, indicating optimal binding relative to its molecular size, while bemcentinib provided a complementary profile by combining strong docking performance with acceptable physicochemical and pharmacokinetic properties. Together, these compounds reflect consistent binding compatibility within the ANXA2 binding environment.

EIF2A demonstrated particularly favorable ligand-binding characteristics, with omilancor achieving both strong binding affinity and high LE, supported by a dense and stable interaction network within the binding pocket. Tapotoclax was also retained due to its strong docking performance and relevant interaction profile, although its lower GI absorption indicates comparatively less favorable pharmacokinetic properties.

In the case of STAM, APTO-253 exhibited the highest LE, reflecting optimal binding relative to its molecular size, while bemcentinib showed the strongest binding affinity among the selected ligands. Omilancor was also retained due to its consistent performance across multiple targets and its balanced physicochemical profile.

Overall, this integrated prioritization strategy identified ligands that satisfy structural, energetic, and pharmacokinetic criteria, resulting in a focused and biologically relevant set of candidate molecules for further investigation. A comprehensive overview of the final prioritized ligands, including docking affinity, LE, and ADME-related properties, is presented in Table 1.

**Table 1.** Final prioritized ligands identified through integrated analysis of docking affinity, ligand efficiency (LE), and ADME-related properties. The table summarizes binding affinities (kcal/mol), LE values, and key physicochemical and pharmacokinetic descriptors.

Molecule	Target	Binding affinity (kcal/mol)	LE	MW	LogP (consensus)	TPSA (Å^2^)	HBD	HBA	RotB	GI absorption	P-gp	Lipinski violations	PAINS alerts
Omilancor	ANXA2	−10.1	0.253	528.56	3.11	123.76	2	6	6	High	Yes	1	0
	EIF2A	−11.5	0.288										
	STAM	−9.3	0.233										
Bemcentinib	ANXA2	−9.4	0.247	506.64	4.53	97.78	5	2	4	High	Yes	2	0
	STAM	−9.6	0.253										
Conivaptan	AHNAK2	−9.4	0.247	498.57	5.01	78.09	2	3	6	High	No	0	0
SYHA1813	ANXA2	−9.4	0.303	410.44	4.73	83.80	3	3	4	High	Yes	1	0
APTO-253	STAM	−9.2	0.329	367.38	4.25	70.25	2	4	1	High	Yes	0	0
Avapritinib	ANXA2	−9.4	0.254	498.56	2.22	106.29	1	7	5	High	Yes	0	0
Tapotoclax	EIF2A	−10.9	0.260	613.21	5.14	93.32	1	5	1	Low	Yes	1	0

To support interpretation and reproducibility of the docking results, a comprehensive summary of top-ranked ligands across key targets is provided in Table S6, which integrates binding affinities, docking score ranges, selected poses (pose 1), key interacting residues, interaction types, and structure/model confidence metrics.

Across all targets, the selected ligands demonstrated consistently strong binding profiles, with EIF2A exhibiting the most favorable interactions, followed by ANXA2 and AHNAK2. Recurrent interaction patterns were observed within defined binding pockets, including Gln^179^, Tyr^180^, and Pro^183^ in AHNAK2; Arg^177^/Arg^178^ and Glu^180^ in ANXA2; Lys^126^–Asn^148^ clusters in EIF2A; and Ile^218^, Ala^233^, and Met^274^ in STAM. These residues were identified as key interaction hotspots based on their recurrence across top-ranked ligands and their involvement in stabilizing interactions, particularly hydrogen bonding and π-mediated contacts.

Of note, docking into AlphaFold structures is most reliable when restricted to confident regions, and our workflow followed this practice. Full structural refinement or intrinsically disordered region (IDR) truncation would require a separate study beyond the scope of this manuscript. Both global (average pLDDT) and local (binding site pLDDT) confidence scores for all AlphaFold-derived models, alongside crystallographic resolution for experimentally determined structures, showed structurally reliable regions. Low-confidence regions were not explicitly removed prior to docking. This decision was made to preserve the full structural context of each protein, as a blind docking strategy was employed to enable unbiased exploration of potential ligand-binding sites across the entire protein surface. Importantly, our docking analyses and subsequent interpretation focused on interactions occurring within structurally confident regions, as supported by the reported binding site pLDDT values.

Structural relaxation was not performed prior to docking. This approach was consistent with standard large-scale virtual screening workflows using AutoDock Vina, where structures are used following standard preparation without additional minimization. Given the comparative nature of this study and the consistent preparation of all protein models, this does not introduce systematic bias in ligand ranking. Docking was conducted using full-length protein structures rather than isolated domains. This choice aligned with the blind docking approach and ensured that no potential binding regions were excluded a priori, which was particularly relevant in the absence of confidently predicted binding pockets. Future studies incorporating structure refinement and targeted binding site definition may further improve prediction accuracy.

It is known that docking free energy alone can favor larger molecules and that LE metrics or ADME filters improve prioritization in medicinal chemistry pipelines. In the present study, however, we established a proteomics-anchored target prioritization framework rather than studied a medicinal chemistry optimization. We showed below how ligand size, physicochemical considerations, and drug-likeness were handled within the scope of this study.

Clarification: Docking Δ*G* values were used solely as a binary filter to indicate the presence of a plausible, stable interaction between a clinically characterized small molecule and a structured protein pocket. Δ*G* was used neither to rank compounds nor to infer therapeutic feasibility. Docking did not aim to optimize hit potency or binding efficiency. All compounds were already prefiltered for clinical relevance. The ligand library (7,509 compounds) consisted exclusively of clinically characterized molecules (approved drugs or agents in clinical phase I/II trials). These molecules already incorporate medicinal chemistry and ADME constraints, including dosing feasibility, molecular weight boundaries, and real-world pharmacokinetics. Therefore, no lead-like filtering or ADME prediction was required at this stage. This justifies the absence of additional drug-likeness filtering in the current framework.

Large molecular scaffolds were not selected because of Δ*G* advantage. Molecules such as zavegepant, bemcentinib, omilancor, and padnarsertib were selected not because they produced lower Δ*G* than smaller molecules but because (a) they met all 5 target criteria; (b) they form chemically interpretable interactions (H-bonds, halogen contacts, π-stacking) with well-structured domains; and (c) they have known clinical pharmacology, reducing concerns about molecular size or permeability. This clarifies that docking Δ*G* merely supported binding plausibility, not drug prioritization.

Because our study was intentionally limited to target prioritization rather than ligand optimization, we did not compute LE metrics. LE-based hit filtering is important during medicinal chemistry refinement but out of scope for the present target-oriented study, which seeks to identify proteins for follow-up validation. The compounds mentioned are illustrative docking exemplars, not proposed as optimized chemical leads.

ADME prediction requires dedicated modeling pipelines and assumptions about formulation, dosing, and PDAC stromal penetration. These were intentionally outside the scope of the present study, whose goal is to provide a protein-centric computational workflow, not a compound-centric optimization pipeline. Because the compound library consisted only of clinically characterized agents, ADME properties are already established and accessible through public registries, enabling downstream work by others. This would maintain appropriate scope boundaries.

Following the initial docking and refinement step, LE values were calculated for the top-ranked ligands per target by normalizing binding affinity to heavy atom count. This allowed comparison of binding efficiency independent of molecular size. The results indicated that several compounds with moderate docking scores exhibited favorable efficiency profiles, such as SYHA1813 (LE = 0.303) and APTO-253 (LE = 0.329), highlighting their potential as efficient binders despite smaller molecular size. In addition, ADME and drug-likeness properties were evaluated for the selected top candidates, including molecular weight, lipophilicity (LogP), TPSA, hydrogen bond donors/acceptors, rotatable bonds, GI absorption, P-gp interaction, Lipinski rule compliance, and PAINS alerts. These results demonstrate that several top-performing ligands exhibit favorable pharmacokinetic profiles, including high GI absorption and limited rule violations

#### Docking energies

Docking free-energy values from AutoDock Vina carry an intrinsic uncertainty (typically 2 to 3 kcal/mol), and therefore, small numerical differences should not be overinterpreted. The present study was not to infer rank-order potency or quantitative superiority of any compound. Instead, the docking energies were used solely to confirm the presence of a plausible, stable protein–ligand interaction within structurally confident pockets.

The clarifications to ensure that docking results were accurately framed were as follows: (a) Differences in docking scores within 2 to 3 kcal/mol should be interpreted cautiously, as they fall within the expected uncertainty of the scoring function. The purpose of reporting these values was descriptive, not inferential. No claims of superior affinity were intended for values within this margin. This aligns our interpretation with accepted docking reproducibility limits. (b) Docking was used to validate that a structured, ligandable pocket exists on the prioritized proteins. Compounds were not prioritized on the basis of Δ*G* magnitude. Docking was one component of a 5-criterion target-centric framework, not a ligand optimization study. Thus, the conclusions should not depend on fine-grained differences in Δ*G*. (c) Given that the present study focuses on target identification, not medicinal chemistry refinement, multiple docking runs, consensus scoring, or ensemble docking would be appropriate for lead optimization, but were beyond the scope of this proteomics-anchored target prioritization pipeline.

#### AHNAK2

Docking analysis of AHNAK2 identified conivaptan as the top prioritized ligand based on its balanced performance across docking affinity, LE, and ADME-related properties (Table 1). Conivaptan demonstrated a stable binding mode characterized by a combination of multiple hydrogen bonds and hydrophobic contacts, forming a well-balanced interaction profile within the predicted binding pocket. The ligand appears to be anchored within a polar region of the pocket while simultaneously engaging surrounding hydrophobic residues, indicating strong complementarity between ligand structure and pocket architecture.

**Fig. 6.**
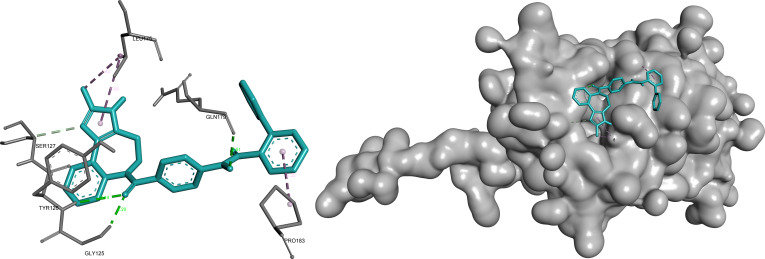
Conivaptan exhibits a balanced polar–hydrophobic binding mode within the AHNAK2 pocket, anchored by multiple hydrogen bonds (green) and reinforced by surrounding hydrophobic contacts (pink/purple). Key interactions include hydrogen bonding with Gly^125^, Tyr^126^, Ser^127^, and Gln^179^ (2.20 to 3.86 Å), alongside hydrophobic contacts with Leu^175^ and Pro^183^ (4.42 to 5.22 Å).

This mixed interaction pattern suggests a favorable binding environment, supporting consistent ligand accommodation and stability. The observed interaction profile aligns with the prioritization of conivaptan as the leading AHNAK2 candidate (Fig. 6).

The high-affinity docking poses we observed for AHNAK2 (conivaptan and related compounds) echoed recent functional studies that nominated AHNAK2 as both biomarker and candidate effector of invasive signaling [40,41]. Targeting AHNAK2, with the aim of destabilizing its interactions that sustain receptor tyrosine kinase signaling, could be a promising angle for combination strategies, particularly where c-MET or related pathways are active. AHNAK2 also stabilizes c-MET signaling [40], consistent with its role in invasive phenotypes.

#### ANXA2

For ANXA2, 3 ligands—omilancor, bemcentinib, and SYHA1813—were prioritized based on integrated evaluation criteria, reflecting the adaptability and structural diversity of the binding pocket.

**Fig. 7.**
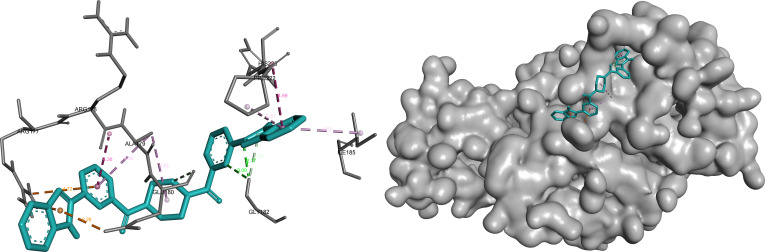
Omilancor forms an extensive and highly interconnected interaction network within ANXA2, dominated by electrostatic (orange) and π-mediated contacts (pink/purple). Prominent interactions include π-cation contacts with Arg^177^ (3.25, 4.72 Å) and hydrogen bonding with Glu^180^ (3.26 Å) and Gly^182^ (2.17 to 3.09 Å), supported by multiple π-alkyl and amide–π interactions stabilizing the ligand.

Omilancor exhibited the most extensive and interconnected interaction network among the selected ligands, combining electrostatic, hydrogen bonding, and π-mediated interactions. The presence of multiple charged and aromatic contacts suggests strong complementarity with the ANXA2 binding environment and supports its role as the primary candidate for this target (Fig. 7).

**Fig. 8.**
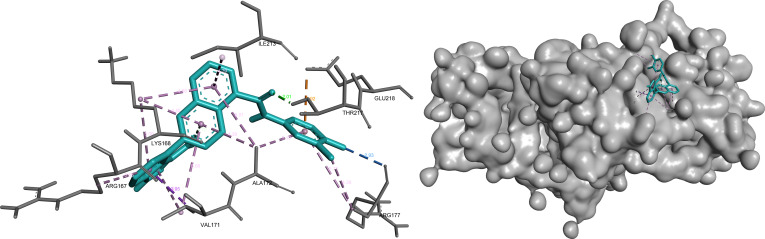
SYHA1813 adopts a compact, hydrophobic-dominated binding mode within ANXA2, with selective polar interactions contributing to specificity. Key contacts include π-alkyl (pink) interactions with Lys^168^, Val^171^, and Ala^172^ (4.39 to 5.66 Å), a hydrogen bond (green) with Thr^217^ (2.01 Å), and additional halogen (blue) and π-anion (orange) interactions enhancing binding stability.

SYHA1813 displayed a more compact binding mode dominated by hydrophobic interactions, with fewer but strategically positioned polar contacts. This interaction pattern aligns with its high LE, indicating effective binding relative to molecular size while maintaining stable accommodation within the pocket (Fig. 8).

**Fig. 9.**
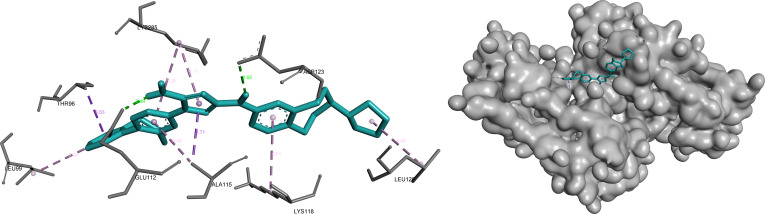
Bemcentinib displays a balanced interaction profile within ANXA2, combining polar anchoring with hydrophobic stabilization. Hydrogen bonds (green) with Glu^112^ (2.49 Å) and Asp^123^ (2.65 Å) are complemented by hydrophobic (pink) and π-sigma (purple) interactions involving Leu^99^, Ala^115^, Lys^118^, and Thr^96^.

Bemcentinib demonstrated a balanced interaction profile, combining polar anchoring interactions with hydrophobic stabilization. This complementary binding behavior, distinct from both omilancor and SYHA1813, further supports the ability of ANXA2 to accommodate structurally diverse ligands within a consistent binding region (Fig. 9).

ANXA2 is a multifunctional, calcium-dependent phospholipid-binding protein with well-documented roles in membrane trafficking, plasminogen activation, cytoskeletal remodeling, and tumor invasiveness. It has been repeatedly linked to metastasis, immune modulation, and therapy resistance across cancers [42,43]. Our observation that ANXA2 binds calcitonin gene-related peptide (CGRP) antagonist scaffolds (e.g., zavegepant) and other high-affinity ligands in silico suggested a structural complementarity that could influence ANXA2’s membrane-associated functions [e.g., exocytosis/endocytosis and soluble N-ethylmaleimide-sensitive factor attachment protein receptor (SNARE) complex regulation]. Considering emerging evidence that manipulating ANXA2 can alter the tumor immune microenvironment, repurposed ligands that affect ANXA2 conformational states might have dual effects on tumor cell behavior and tumor immunity in PDAC.

Given its role in exocytosis, plasminogen activation, and immune modulation, these compounds may perturb PDAC’s communication and invasion machinery, which is consistent with [8,9].

#### EIF2A

EIF2A demonstrated one of the most favorable binding environments among the analyzed targets, with omilancor and tapotoclax identified as the top candidates.

**Fig. 10.**
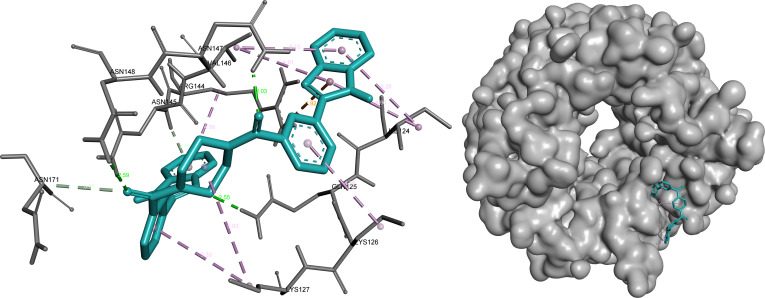
Omilancor exhibits a dense and highly stabilized binding network within EIF2A, characterized by extensive hydrogen bonding (green) and electrostatic interactions (orange). Key interactions include hydrogen bonds with Gln^125^, Asn^145^, Asn^147^, and Asn^148^ (2.03 to 2.59 Å), alongside π-mediated (pink/purple) interactions with Arg^144^, Lys^126^, and Lys^127^ residues.

Omilancor formed a dense and highly stabilized interaction network characterized by multiple hydrogen bonds and π-mediated interactions. The combination of polar anchoring and electrostatic stabilization indicates strong binding complementarity and suggests a highly optimized fit within the EIF2A binding pocket (Fig. 10).

**Fig. 11.**
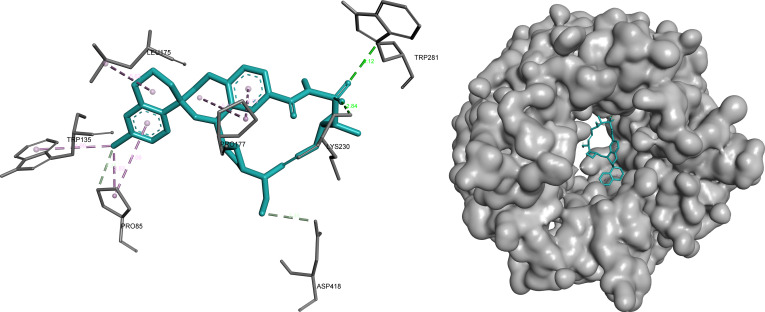
Tapotoclax adopts a more localized binding mode within EIF2A, forming a compact interaction network supported by hydrogen bonding (green) and hydrophobic contacts (pink/purple). Notable interactions include hydrogen bonds with Pro^85^, Lys^230^, and Asp^418^ (2.81 to 3.38 Å), and π-alkyl contacts with Trp^135^ and Pro^177^.

In contrast, tapotoclax displayed a more localized binding mode, supported by a combination of hydrogen bonding and hydrophobic interactions. This more compact interaction profile indicates effective ligand accommodation within a defined region of the pocket, albeit with lower interaction complexity compared to omilancor (Fig. 11).

EIF2A is increasingly implicated in noncanonical initiation and ribosomal subunit homeostasis during stress conditions. Recent studies show that it can regulate 40S subunit behavior and influence translation re-initiation under stress [44,45]. Our docking results had very favorable energies to padnarsertib, omilancor, and other scaffolds and therefore raised the possibility of modulating stress-responsive translational control in PDAC via small molecules that bind EIF2A’s functional surface. Given EIF2A’s association with poorer prognosis in PDAC cohorts, perturbation of EIF2A activity could plausibly alter tumor cell adaptation to metabolic and therapeutic stress. EIF2A regulates stress-responsive translation, linking it to gemcitabine resistance and metabolic adaptation [7]

#### STAM

In STAM, bemcentinib, omilancor, and APTO-253 were retained following integrated prioritization, all occupying a consistent binding region defined by key residues of the predicted pocket.

**Fig. 12.**
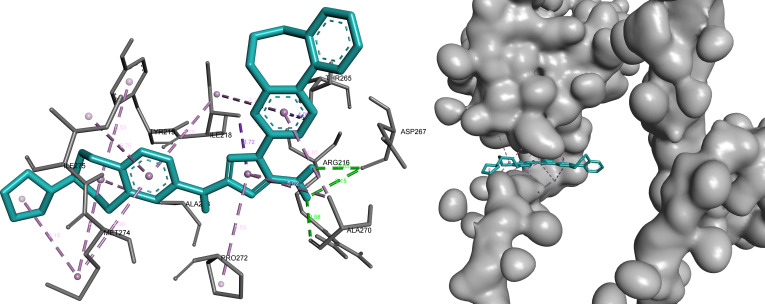
Bemcentinib demonstrates a stable binding mode within STAM, combining polar interactions with hydrophobic stabilization. Key interactions include hydrogen bonds (green) with Asp^267^ and Ala^270^ (2.55 to 3.15 Å), supported by π-sigma (purple) and hydrophobic contacts (pink) involving Ile^218^, Thr^265^, Ala^233^, and Met^274^.

Bemcentinib exhibited the strongest binding characteristics, combining hydrogen bonding and hydrophobic interactions that support stable ligand positioning within the pocket. This interaction pattern reflects strong complementarity with the binding environment and reinforces its prioritization for this target (Fig. 12).

**Fig. 13.**
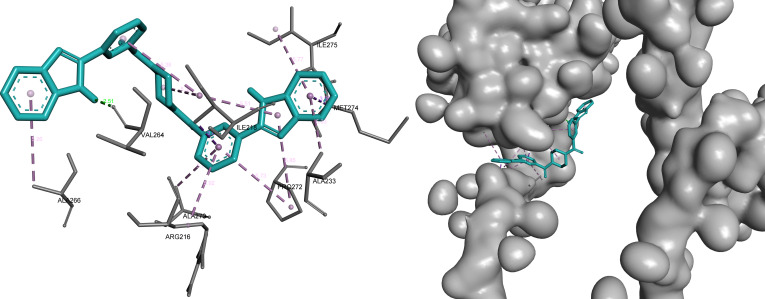
Omilancor maintains a consistent binding orientation within the STAM pocket, with interactions dominated by π-mediated and hydrophobic contacts. These include π-sigma interactions with Ile^218^ and Met^274^ (purple), hydrogen bonding (green) with Val^264^ (2.51 Å), and multiple π-alkyl interactions (pink) stabilizing the ligand.

Omilancor showed a similar binding orientation, maintaining a consistent interaction pattern within the same pocket region. Its interactions are primarily stabilized through hydrophobic and π-mediated contacts, supporting reliable and reproducible binding behavior across targets (Fig. 13).

**Fig. 14.**
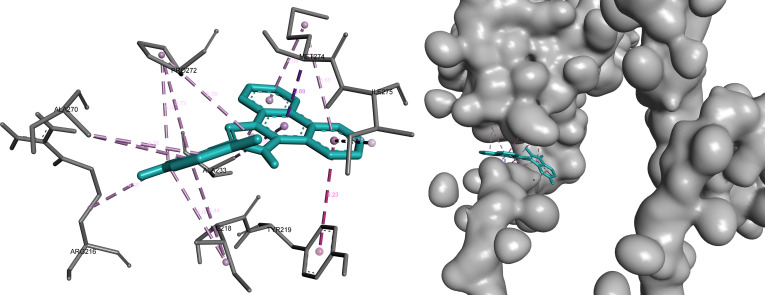
APTO-253 exhibits a hydrophobic and aromatic interaction-dominated binding mode within STAM, consistent with its high ligand efficiency. The ligand engages key residues through π–π stacking (magenta), π-sigma (purple), and hydrophobic contacts (pink), forming an extensive stabilization network.

APTO-253 demonstrated the highest LE and formed an interaction network dominated by hydrophobic and aromatic contacts. This profile indicates efficient binding relative to molecular size and highlights a distinct yet compatible interaction mode within the STAM binding site (Fig. 14).

STAM is a key component of the endosomal sorting complexes required for transport-0 (ESCRT-0) complex, which mediates ubiquitin-dependent cargo recognition and sorting in the endosomal pathway, thereby contributing to receptor trafficking, degradation, and extracellular vesicle biogenesis. Aberrations in endosomal/ESCRT biology can modulate surface expression of oncogenic receptors and immune checkpoints and thereby affect signaling amplitude and immune recognition [46–48]. Our docking hits for STAM (with bemcentinib and umbralisib) revealed a potential ligand-accessible pocket that could represent a structural vulnerability within the ESCRT-0 subunit. Such interactions could conceivably be leveraged to modulate receptor trafficking or alter extracellular vesicles’ cargo loading in PDAC, consistent with STAM’s role in ubiquitin-dependent endosomal sorting and extracellular vesicle biogenesis. Given the intimate link between endosomal sorting, PD-L1 dynamics, and intercellular communication, modulation of STAM-related activities may have broader effects on tumor–microenvironment crosstalk [49]. Because STAM is central to ESCRT-0, these findings tie directly to exosomal mediation of immune evasion [8] and PD-L1 trafficking.

#### CGRP hypothesis

Recent advances in cancer neuroscience have highlighted that sensory neurons actively shape tumorigenesis [50]. A very recent study demonstrated that nociceptor-derived CGRP promotes PDAC progression by establishing the CGRP–cancer-associated fibroblasts (CAF)–natural killer (NK) axis [51]. In the present study, we identified structurally ligandable pockets in EIF2A, STAM, ANXA2, and AHNAK2, with several clinically validated compounds ranking among the strongest docking candidates. Notably, zavegepant, a clinically approved CGRP receptor antagonist, emerged as a high-affinity ligand through unbiased docking. Its identification was driven not by prior mechanistic assumptions but by structural complementarity observed during blind docking. While none of the 4 prioritized proteins participate in canonical CGRP receptor signaling, the intersection between these computational findings and the newly uncovered CGRP-dependent neuro-immune axis raises the possibility that PDAC may harbor noncanonical pockets capable of accommodating CGRP-active chemical scaffolds.

It should be kept in mind that our results do not imply that zavegepant or related scaffolds modulate CGRP signaling in PDAC, nor that these proteins interact with CALCRL/RAMP1. Instead, our findings highlight a previously unappreciated chemical convergence, wherein ligands designed for neuropeptide receptor pharmacology also exhibit affinity for PDAC-associated proteins. This suggests a potential avenue for exploring polypharmacology or off-target interactions within PDAC cells.

Taken together, integrating molecular networking, ligandable pocket discovery, and insights from CGRP-mediated regulation highlights a novel exploratory direction: investigating whether CGRP-active compounds possess broader functional relevance in PDAC biology through in silico, in vitro, and in vivo validation studies aimed at repositioning clinically tested drugs toward novel PDAC therapeutic targets [15].

Several high-scoring small molecules that emerged from our screen already have clinical or preclinical pedigrees. Zavegepant (a clinically approved CGRP receptor antagonist) has well-characterized safety data in humans, bemcentinib (AXL inhibitor) and padnarsertib (investigational dual PAK4–NAMPT inhibitor in early-phase) have undergone oncology trials, and omilancor (immune/metabolic modulator under clinical development) and other immune/metabolic modulators have distinct mechanisms that could intersect with PDAC [52–55]. Repurposing these agents for PDAC will require further mechanistic studies to demonstrate that binding to the nominated PDAC protein leads to functional perturbation relevant to cancer biology, rather than off-target affinity. The availability of clinical safety/PK data for many of these agents makes them attractive starting points for rapid translational testing, but regulatory and pharmacodynamic considerations remain.

In general, these 4 proteins belong to pathways corroborated by the 5 external studies, including membrane trafficking and ESCRT signaling [7,8], cytoskeletal reshaping and EMT ([9], immune evasion and stromal crosstalk [7,8], and prognostic subnetworks [10]. GETdb annotations confirmed conserved allosteric pockets for all the 4 proteins. To situate this framework within current PDAC biology, we integrate insights from recent studies, showing (a) complement-driven gemcitabine resistance via C3a/C3aR signaling [7], (b) exosome-mediated intercellular communication as a driver of immune evasion and biomarkers such as miR 1293 [8], (c) TGF-β1/Smad4-driven Hedgehog Gli1 EMT regulation [9], (d) machine learning (ML) integrated network biomarker discovery [10], and (e) the GETdb resource for genetic/evolutionary target characterization [11].

### Clinical association by survival analysis

Clinical significance was assessed using HPA Kaplan–Meier data of 176 PDAC patients (median follow-up 1.27 years) (Table 2). Among the 32 possible druggable proteins, EIF2A, STAM, ANXA2, and AHNAK2 showed log-rank *P* < 0.001, with higher expression associated with poorer overall survival. These associations converged with their network centrality and pathway placement, collectively supporting their prioritization as clinically relevant nodes in PDAC. EIF2A, STAM, ANXA2, and AHNAK2 were all interesting candidates as predictors of poor survival, which could be independent validations for biological and clinical impact.

**Table 2.** HPA survival analysis of 176 PDAC patients (median follow-up 1.27 years) showed that EIF2A, STAM, ANXA2, and AHNAK2 were strong prognostic biomarkers (*P* < 0.001), i.e., high expression with low 5-year survival and vice versa

Protein	Median expression	*P* value	High expression with 5-year survival (%)	Low expression with 5-year survival (%)
EIF2A	51.77	0.000083	11	41
STAM	17.2	0.00080	14	43
ANXA2	1995.77	0.000014	11	43
AHNAK2	45.17	0.000066	20	54

Of note, the survival modeling in PDAC requires careful attention to clinical covariates, follow-up duration, and validation cohorts. It should be noticed that the goal of the present study was not to establish independent prognostic biomarkers or build clinical risk models but to provide a proteomics-anchored target prioritization workflow. The survival analysis as in Table 2 was intended as a supporting descriptive layer to contextualize the 4 prioritized proteins that need to be validated as potential prognostic biomarkers.

It should also be noted that the HPA Kaplan–Meier analysis was univariate and did not adjust for confounders such as tumor stage, grade, age, or treatment, and that the HPA automatically uses median expression split for Kaplan–Meier grouping, with no alternative cutoffs available within the platform. The HPA-PAAD had short follow-up and was not designed for multivariate survival modeling. A multivariate Cox regression model was not included because the present study focuses on molecular target prioritization, not prognostic model development. It would be of interest to perform a multivariate Cox analysis if such data of full clinical covariates are available. However, the 4 proteins (EIF2A, STAM, ANXA2, and AHNAK2) show lower survival in HPA-PAAD only as observational correlates, which must be interpreted cautiously.

## Conclusions

In this study, we present a proteomics-anchored, multimodal knowledge discovery framework that integrates cross-model proteomics harmonization, network topology, structural modeling, and in silico docking to systematically prioritize druggable targets in PDAC. From 1,975 shared proteins, we identified 32 PDAC-expressed candidates, ultimately nominating EIF2A, STAM, ANXA2, and AHNAK2 based on convergent structural, network, and clinical criteria.

We propose that structural filtering, docking validation, LE scoring, and ADME evaluation can strengthen the robustness of the workflow and reveal ligandable pockets with high-affinity interactions involving clinically characterized compounds, highlighting clear opportunities for drug repurposing. Furthermore, we suggest a systems-level resource that identifies experimentally tractable targets and small-molecule opportunities, laying a foundation for future mechanistic and translational studies in PDAC.

## Data Availability

All data needed to evaluate the conclusions of this study are available in the paper and the Supplementary Materials.
