# Supplementary material for: Knowledge Discovery and Drug-Repurposing Framework for Pancreatic Ductal Adenocarcinoma: Molecular Networking and Computational Docking
Source: Comput Struct Biotechnol J. 2026 May 5;35(1):0067. doi: 10.34133/csbj.0067 (PMC13139727; doi:10.34133/csbj.0067)
Supplement: Supplementary 1 — Figs. S1 to S28 Tables S1 to S6 [file csbj.0067.f1.zip › Supplementary Tables S1-S6.pdf]

Supplementary Table S1. Summary statistics for the global, first-neighbor filtered, and MCL-clustered PDAC interaction networks

The global STRING-derived PDAC protein–protein interaction network was constructed from the common PDAC protein set after identifier harmonization in Cytoscape. A first-neighbor filtered subnetwork containing the 32 docking-selected proteins and their directly connected interaction partners was then extracted from the global network. The same first-neighbor node set was subsequently visualized after Markov Cluster Algorithm (MCL) clustering, resulting in a reduced edge count in the clustered representation.

Network level	Description	Nodes (n)	Edges (n)
Global PDAC interactome	STRING-derived network constructed from the common PDAC protein set after identifier harmonization	1973	74582
First-neighbor filtered network	Subnetwork containing the 32 docking-selected proteins and their directly connected interaction partners	857	26155
MCL-clustered first-neighbor network	Clustered representation of the first-neighbor filtered network used for module-level visualization	857	9270

Supplementary Table S2. Cluster assignment of docking-selected proteins in the first-neighbor PDAC interaction network

Docking-selected proteins were mapped onto the first-neighbor filtered PDAC interaction network and assigned to MCL modules where applicable. For each protein, the table reports the MCL cluster number, module size, degree in the clustered network, module status, and corresponding Figure 5 panel. Proteins without an assigned MCL cluster appeared as singletons in the clustered network and are summarized in Figure 5P.

Docking-selected protein	MCL cluster	Module size (n)	Degree in clustered network	Status	Figure 5 panel
ACADSB	1	137	56	Multi-node module	A
PCCA	1	137	49	Multi-node module	A
PCK2	1	137	32	Multi-node module	A
PDHB	1	137	96	Multi-node module	A
ACTR1A	2	91	34	Multi-node module	B
GSN	2	91	40	Multi-node module	B
MYH14	2	91	11	Multi-node module	B
SH3BGRL3	2	91	4	Multi-node module	B
EIF2A	3	70	44	Multi-node module	C
TIMM50	6	24	17	Multi-node module	D
POLR2H	9	19	17	Multi-node module	E
SNAP23	10	17	14	Multi-node module	F
VTI1B	10	17	16	Multi-node module	F
ATP6V1F	11	17	16	Multi-node module	G
MVB12A	13	15	14	Multi-node module	H
STAM	13	15	13	Multi-node module	H
AHNAK2	24	8	2	Multi-node module	I
ANXA2	24	8	7	Multi-node module	I
YWHAQ	26	7	6	Multi-node module	J
SPTAN1	36	5	4	Multi-node module	K
SCO1	46	3	1	Multi-node module	L
SURF1	46	3	2	Multi-node module	L
PACSIN2	52	2	1	Multi-node module	M
MYO1C	58	2	1	Multi-node module	N
OTUB1	64	2	1	Multi-node module	O
ANXA3	–	–	0	Singleton	P
HPCAL1	–	–	0	Singleton	P
PLP2	–	–	0	Singleton	P
SCPEP1	–	–	0	Singleton	P

Docking-selected protein	MCL cluster	Module size (n)	Degree in clustered network	Status	Figure 5 panel
SERPINB6	–	–	0	Singleton	P
SFXN2	–	–	0	Singleton	P
VIL1	–	–	0	Singleton	P

Supplementary Table S3. MCL modules containing docking-selected proteins in the first-neighbor PDAC interaction network

This table summarizes all non-singleton MCL clusters that contained one or more docking-selected proteins in the clustered first-neighbor PDAC interaction network. For each module, the cluster number, total module size, number of docking-selected proteins, docking-selected protein identities, and corresponding Figure 5 panel are listed. Modules are ordered numerically by MCL cluster identifier. Docking-selected proteins that remained unclustered are listed separately as singletons.

MCL cluster	Module size (n)	No. of docking-selected proteins	Docking-selected proteins in module	Figure 5 panel
1	137	4	ACADSB, PCCA, PCK2, PDHB	A
2	91	4	ACTR1A, GSN, MYH14, SH3BGRL3	B
3	70	1	EIF2A	C
6	24	1	TIMM50	D
9	19	1	POLR2H	E
10	17	2	SNAP23, VTI1B	F
11	17	1	ATP6V1F	G
13	15	2	MVB12A, STAM	H
24	8	2	AHNAK2, ANXA2	I
26	7	1	YWHAQ	J
36	5	1	SPTAN1	K
46	3	2	SCO1, SURF1	L
52	2	1	PACSIN2	M
58	2	1	MYO1C	N
64	2	1	OTUB1	O
Singleton candidates	7	7	ANXA3, HPCAL1, PLP2, SCPEP1, SERPINB6, SFXN2, VIL1	P

Supplementary Table S4. Structural quality assessment of protein models used for docking. Experimentally determined structures are reported with crystallographic resolution, while AlphaFold-predicted models are evaluated using both global (average pLDDT) and local (binding-site pLDDT) confidence metrics.

Protein	Structure Source	PDB ID / AlphaFold ID	Resolution (Å)	Avg pLDDT	Binding Site pLDDT
ACADSB	PDB	2JIF	2.00	-	-
ACTR1A	AlphaFold	AF-P61163-F1-v6	-	92.94	95.87
AHNAK2	PDB	4CN0	1.75	-	-
ANXA2	PDB	2HYU	1.86	-	-
ANXA3	PDB	1AII	1.95	-	-
ATP6V1F	PDB	6WM2	3.10	-	-
EIF2A	PDB	8DYS	1.80	-	-
GSN	PDB	3FFN	3.00	-	-
HPCAL1	AlphaFold	AF-P37235-F1-v6	-	86.75	83.12
MVB12A	PDB	6VME	2.19	-	-
MYH14	PDB	5JLH	3.90	-	-
MYO1C	PDB	4BYF	2.74	-	-
OTUB1	PDB	2ZFY	1.69	-	-
PACSIN2	PDB	3ABH	2.00	-	-
PCCA	PDB	7YBU	2.20	-	-
PCK2	AlphaFold	AF-Q16822-F1-v6	-	93.94	94.54
PDHB	PDB	3EXE	1.98	-	-
PLP2	AlphaFold	AF-Q04941-F1-v6	-	78.75	90.36
POLR2H	PDB	7OB9	2.70	-	-
SCO1	PDB	2GGT	2.40	-	-
SCPEP1	AlphaFold	AF-Q9HB40-F1-v6	-	90.12	89.46
SERPINB6	AlphaFold	AF-P35237-F1-v6	-	90.56	79.61
SFXN2	AlphaFold	AF-Q96NB2-F1-v6	-	85.00	85.57
SH3BGRL3	AlphaFold	AF-Q9H299-F1-v6	-	96.25	96.15
SNAP23	AlphaFold	AF-O00161-F1-v6	-	82.12	84.86
SPTAN1	PDB	3F31	2.30	-	-
STAM	AlphaFold	AF-Q92783-2-F1-v6	-	77.19	73.76
SURF1	AlphaFold	AF-Q15526-F1-v6	-	81.38	85.12
TIMM50	PDB	4QQF	2.67	-	-
VIL1	AlphaFold	AF-P09327-F1-v6	-	77.31	75.11
VTI1B	PDB	2V8S	2.22	-	-
YWHAQ	PDB	6BD2	2.90	-	-

Supplementary Table S5: Comprehensive summary of protein–ligand interaction profiles obtained from in silico docking across all analyzed targets. The table includes top-ranked ligands for each protein, their corresponding binding affinities (kcal/mol), and detailed interaction patterns, including hydrogen bonds, hydrophobic contacts (alkyl and π-alkyl), π–π stacking, π-cation/π-anion interactions, halogen bonding, and π-sigma interactions. Interaction distances (Å) are provided to support structural interpretation. The results demonstrate diverse binding modes across targets, reflecting variability in binding pocket architecture and ligand physicochemical complementarity. Representative interactions highlight recurrent stabilization patterns involving key residues within functionally relevant regions, while comparison with the reference compound gemcitabine reveals generally reduced interaction complexity and weaker binding profiles.

Protein	Ligand	Binding affinity (kcal/mol)	Interactions
ACADSB	Hypericin (CID: 3663)	-13.7	Ser177 hydrogen (2.12 Å), Trp207 pi-alkyl (4.58, 4.99 Å), pi-pi stacked (3.72, 3.77, 3.93, 4.30, 4.36, 4.37, 4.41, 4.43, 4.62, 4.76, 4.79, 5.48, 5.54, 5.64 Å), Lys252 pi-alkyl (5.24 Å), Ile409 pi-sigma (3.63 Å), Ile412 alkyl (5.77 Å), pi-alkyl (5.25, 5.37, 5.45 Å), Tyr413 pi-pi t-shaped (5.30 Å)
	Conivaptan (CID: 151171)	-13.4	Ser177 hydrogen (3.15 Å), Ser209 hydrogen (2.33 Å), Lys252 pi-alkyl (4.16, 5.12, 5.99 Å), Leu253 pi-sigma (3.79 Å), Thr260 pi-sigma (3.71 Å), Ile409 pi-alkyl (4.51, 5.33 Å)
	Adavivint (CID: 135565709)	-13.3	Glu140 halogen (3.16 Å), Phe174 pi-alkyl (5.80 Å), Leu176 alkyl (5.04 Å), Trp207 pi-sigma (3.70, 3.75 Å), pi-pi stacked (4.26, 4.85, 5.28, 5.39 Å), Ser209 hydrogen (2.12 Å), Lys252 hydrogen (3.13 Å), pi-alkyl (5.85 Å), Leu290 alkyl (4.38 Å), Ile409 pi-sigma (3.68 Å), Ile412 alkyl (5.37 Å), Glu414 halogen (3.66 Å)
	Lumacaftor (CID: 16678941)	-13.1	Gly182 hydrogen (2.48 Å), Ser183 hydrogen (2.76 Å), Trp207 pi-pi stacked (3.71, 3.89 Å), Ser209 hydrogen (3.13 Å), Lys252 pi-alkyl (5.96 Å), Thr260 hydrogen (3.49 Å), pi-sigma (3.81 Å), Ile287 alkyl (4.59 Å), Leu290 pi-alkyl (5.90 Å), Arg294 hydrogen (2.19 Å), Ile409 pi-sigma (3.99 Å), Ile412 pi-alkyl (5.40 Å), Tyr413 pi-pi t-shaped (4.91 Å), Glu414 hydrogen (3.08 Å)

	LUT-014 (CID: 138319775)	-13.0	Ile141 alkyl (3.89 Å), Leu145 alkyl (5.61 Å), Phe174 hydrogen (2.62 Å), halogen (3.52 Å), pi-alkyl (5.62 Å), Trp207 hydrogen (3.17 Å), pi-pi stacked (4.41, 4.65, 5.92 Å), pi-alkyl (4.35 Å), Ser209 hydrogen (2.75, 2.99, 3.32 Å), halogen (3.38 Å), Lys252 pi-alkyl (3.17, 3.60 Å), Leu253 pi-sigma (3.86, 3.94 Å), Thr260 hydrogen (3.53 Å), pi-sigma (3.80 Å), Lys408 pi-alkyl (4.97 Å), Ile409 hydrogen (3.16 Å), pi-alkyl (4.29, 5.31, 5.42 Å), pi-sigma (3.88 Å), Ile412 pi-alkyl (4.90, 5.34, 5.80 Å), Tyr413 pi-pi t-shaped (5.30 Å)
	Gemcitabine (CID: 60750)	-7.4	Gly415 hydrogen (1.89 Å)
ACTR1A	Bifeprunox (CID: 208951)	-11.4	Asp15 pi-anion (4.91 Å), Val20 alkyl (4.78 Å), Lys22 hydrogen (2.53 Å), Asp159 hydrogen (2.71 Å), pi-anion (4.31 Å), Glu219 pi-anion (3.93 Å), Gly302 hydrogen (2.67 Å), Gly303 hydrogen (3.21 Å), Leu306 pi-alkyl (4.76, 5.64 Å), Phe307 pi-pi t-shaped (5.03 Å), Leu337 alkyl (5.56 Å), pi-sigma (3.86 Å), Thr340 hydrogen (2.73, 2.98 Å)
	Canagliflozin (CID: 24812758)	-11.3	Asp15 hydrogen (3.93 Å), Gly17 hydrogen (3.26 Å), Val20 alkyl (4.07 Å), pi-alkyl (4.48 Å), Lys22 hydrogen (2.33 Å), Gln142 hydrogen (2.83 Å), Gly161 hydrogen (3.32 Å), Asp162 hydrogen (2.22 Å), Glu219 pi-anion (3.78 Å), halogen (3.06 Å), Ser301 hydrogen (3.71 Å), Gly303 amide-pi stacked (3.62 Å), Ser304 amide-pi stacked (3.62 Å), Leu306 pi-alkyl (4.65 Å), halogen (3.35 Å), Phe307 pi-pi t-shaped (5.12 Å), Arg336 hydrogen (3.98 Å), Leu337 hydrogen (2.26 Å), Thr340 hydrogen (3.07 Å)
	Hypericin (CID: 3663)	-11.0	Lys218 hydrogen (3.89 Å), Glu219 hydrogen (2.95 Å), pi-anion (3.36, 3.84, 3.94, 4.18, 4.57, 4.93 Å), Gly303 amide-pi stacked (3.62 Å), Ser304 amide-pi stacked (3.62 Å), Leu306 pi-alkyl (4.80, 4.86, 4.97, 5.11 Å), pi-sigma (3.70 Å), Phe307 pi-pi t-shaped (4.96 Å), Leu337 alkyl (4.89 Å), pi-alkyl (4.74, 5.75, 5.83 Å), pi-sigma (3.41, 3.75 Å)

	Vebreltinib (CID: 72202701)	-10.8	Asp15 hydrogen (3.80 Å), Gly19 halogen (3.69 Å), Val20 hydrogen (4.30 Å), pi-alkyl (4.58 Å), Asp162 halogen (3.77 Å), Lys218 pi-alkyl (5.66 Å), Glu219 halogen (3.16 Å), pi-anion (4.06, 4.12 Å), Gly303 hydrogen (3.45 Å), amide-pi stacked (3.39, 3.85 Å), Ser304 hydrogen (3.14 Å), amide-pi stacked (3.39, 3.85 Å), Leu306 alkyl (4.41 Å), pi-sigma (3.99 Å), Phe307 pi-pi t-shaped (5.37 Å)
	Cirtuvivint (CID: 132056570)	-10.8	Gly19 hydrogen (3.24 Å), Val20 pi-sigma (3.59 Å), Asp162 pi-anion (4.59 Å), pi-sigma (3.76 Å), Glu219 hydrogen (3.57 Å), Leu306 pi-alkyl (4.55, 4.97, 5.29 Å), Phe307 pi-pi t-shaped (5.08, 5.32 Å)
	Gemcitabine (CID: 60750)	-7.4	Gly17 hydrogen (2.98 Å), Ser18 hydrogen (2.90, 3.37 Å), Val20 pi-alkyl (5.36 Å), Gln142 hydrogen (2.89, 3.49 Å), halogen (2.90 Å), Asp159 hydrogen (2.68 Å), halogen (2.53, 2.95 Å), Gly161 hydrogen (3.30 Å), Val164 hydrogen (2.32, 2.48 Å), His166 hydrogen (3.61 Å), Gly302 hydrogen (3.53 Å)
AHNAK2	Lumacaftor (CID: 16678941)	-9.6	Ala123 hydrogen (2.72), Gln179 hydrogen (2.22), Tyr180 hydrogen (2.62), pi-alkyl (5.41), pi-pi stacked (4.17), Glu182 pi-anion (4.05), Pro183 alkyl (4.53)
	Conivaptan (CID: 151171)	-9.6	Gly125 hydrogen (2.20), Tyr126 hydrogen (3.14), Ser127 hydrogen (3.86), Leu175 alkyl (4.42), pi-alkyl (5.09), Gln179 hydrogen (2.51), Pro183 pi-alkyl (5.22)
	Omipalisib (CID: 25167777)	-9.4	Ser124 hydrogen (3.55), Ser127 halogen (3.53), Val128 hydrogen (2.15), pi-alkyl (5.44), halogen (3.90), Asp173 hydrogen (3.59), Leu175 pi-alkyl (5.22), Gln179 hydrogen (3.75), Tyr180 pi-pi stacked (3.81), Pro183 pi-alkyl (5.38, 5.46)
	MK-0773 (CID: 11950726)	-9.3	Val141 hydrogen (2.06, 2.14), alkyl (4.28), Leu142 pi-alkyl (5.35), Lys143 alkyl (5.04), halogen (3.49), Ala148 alkyl (3.67, 4.07, 5.24), Arg154 alkyl (4.92), Glu155 hydrogen (3.41)
	Tuspetinib (CID: 135390910)	-9.2	Tyr126 pi-alkyl (4.09), pi-pi T-shaped (5.20), Val128 alkyl (3.65), Asp173

			hydrogen (2.33), Leu175 alkyl (5.47), pi-alkyl (5.56), Lys176 alkyl (5.21), Leu178 hydrogen (2.59), alkyl (4.66), pi-alkyl (5.51), Gln179 hydrogen (3.08), pi-sigma (3.73), Tyr180 pi-alkyl (5.14, 5.77), pi-pi T-shaped (5.24), Pro183 alkyl (4.23)
	Gemcitabine (CID: 60750)	-5.6	Ala123 hydrogen (2.23 Å), Gly125 hydrogen (3.16 Å), Tyr126 hydrogen (3.39 Å), halogen (3.46 Å), Gln179 hydrogen (2.35 Å)
ANXA2	Zavegepant (CID: 53472683)	-10.2	Arg178 hydrogen (3.75), Asp181 pi-anion (5.00), Val221 hydrogen (2.27), alkyl (3.59), pi-alkyl (5.84), Pro222 hydrogen (3.86), alkyl (5.13), Asn264 hydrogen (3.35), Lys265 hydrogen (2.04), pi-alkyl (5.69), Pro266 alkyl (4.28, 5.06), Arg294 pi-cation (4.67), Val297 alkyl (4.89), pi-alkyl (5.69, 5.94), Asp298 pi-anion (4.07), Lys301 alkyl (4.02, 5.06), pi-alkyl (5.76)
	Omilancor (CID: 121299620)	-10.1	Arg177 pi-cation (3.25, 4.72), Arg178 amide-pi stacked (4.36), Ala179 alkyl (4.82), amide-pi stacked (4.36), pi-alkyl (4.92), Glu180 hydrogen (3.26), pi-anion (3.28), Gly182 hydrogen (2.17, 2.84, 3.09), Ile185 pi-alkyl (4.83), Pro222 amide-pi stacked (4.69), pi-alkyl (5.51), His223 amide-pi stacked (4.69)
	Avapritinib (CID: 118023034)	-9.4	Asp125 hydrogen (3.42), Leu240 alkyl (5.40), pi-alkyl (5.66, 5.86), Arg244 hydrogen (2.61), Leu256 alkyl (5.56), Asn257 hydrogen (3.51), Gln260 halogen (3.33), Gln263 halogen (3.79), Asn264 halogen (3.89), Leu267 pi-sigma (3.67), Asp271 hydrogen (3.57), Val286 alkyl (4.02)
	SYHA1813 (CID: 118331338)	-9.4	Arg167 pi-alkyl (5.55), Lys168 pi-alkyl (4.48, 4.86, 5.09), Val171 pi-alkyl (5.40, 5.66), pi-sigma (3.95), Ala172 pi-alkyl (4.39, 5.01, 5.13), Arg177 alkyl (4.39), pi-alkyl (5.72), halogen (2.93), Ile213 pi-alkyl (4.79), Thr217 hydrogen (2.01), Glu218 pi-anion (3.92)
	Bemcentinib (CID: 46215462)	-9.4	Thr96 pi-sigma (3.63), Leu99 pi-alkyl (5.94), Glu112 hydrogen (2.49), Ala115 pi-alkyl (4.48), pi-sigma (3.71), Lys118 pi-alkyl (4.81), Leu120 alkyl (4.44), Asp123 hydrogen (2.65), Lys285 pi-alkyl (4.11, 5.39)

	Gemcitabine (CID: 60750)	-6.5	Gln68 hydrogen (2.06 Å), Ala71 hydrogen (2.50 Å), pi-sigma (3.65 Å), Leu82 hydrogen (2.71 Å), Ala83 hydrogen (1.85 Å), Leu102 halogen (3.15, 3.32 Å), Gln107 hydrogen (1.89, 2.01 Å)
ANXA3	HRS-4642 (CID: 171853615)	-11.3	Phe207 pi-pi stacked (3.84, 4.16 Å), Pro208 hydrogen (3.30 Å), Cys247 halogen (3.77 Å), Asn250 halogen (3.36 Å), Thr251 hydrogen (3.05 Å), Pro252 hydrogen (3.48 Å), alkyl (4.70, 5.74 Å), pi-alkyl (5.67 Å), Ile283 alkyl (5.27 Å)
	MRTX1133 (CID: 156124857)	-11.1	His9 pi-alkyl (4.35 Å), Arg121 hydrogen (2.53 Å), Phe207 pi-pi stacked (3.85, 4.16 Å), Pro208 hydrogen (3.39 Å), Cys247 halogen (3.80 Å), Asn250 halogen (3.40 Å), Thr251 hydrogen (3.05 Å), Pro252 hydrogen (3.50 Å), alkyl (5.13, 5.20 Å), pi-alkyl (5,58 Å), Ile283 hydrogen (3.41 Å), Asp287 hydrogen (3.99 Å)
	AZD-5991 (CID: 131634760)	-10.9	Ile5 hydrogen (3.69 Å), Val7 hydrogen (3.39 Å), Gln125 hydrogen (2.13 Å), Glu167 hydrogen (2.83 Å), Ser168 hydrogen (3.60 Å), Phe207 pi-pi stacked (3.92, 4.54 Å)
	Danicopan (CID: 118323590)	-10.8	Leu160 alkyl (4.52 Å), Arg165 hydrogen (3.13 Å), alkyl (4.78 Å), pi-alkyl (4.34 Å), pi-cation (4.52 Å), Phe207 hydrogen (2.15 Å), pi-pi stacked (3.92, 4.81 Å), Pro208 hydrogen (3.38 Å), pi-alkyl (5.86 Å), Lys211 hydrogen (2.77 Å), Pro252 alkyl (4.99 Å), Arg280 pi-cation (4.06 Å), Ile283 alkyl (4.25 Å), pi-alkyl (4.78 Å)
	Hypericin (CID: 3663)	-10.7	Arg121 hydrogen (2.59 Å), Ser123 hydrogen (2.40 Å), Arg165 pi-alkyl (5.32 Å), Asp166 hydrogen (1.86 Å), Ser168 hydrogen (2.08 Å), Leu169 pi-alkyl (5.53 Å), Phe207 pi-alkyl (5.34 Å), pi-pi stacked (4.24, 4.30 Å), Pro208 pi-alkyl (4.09, 4.73, 5.32 Å), Lys211 pi-cation (4.29, 4.73 Å)
	Gemcitabine (CID: 60750)	-6.3	His83 hydrogen (1.96, 2.93), Lys98 hydrogen (1.96), pi-alkyl (4.57), Lys102 pi-alkyl (4.72)
ATP6V1F	Atogepant (CID: 72163100)	-9.3	Arg53 hydrogen (2.98, 3.41 Å), alkyl (4.28 Å), pi-alkyl (4.75 Å), halogen (3.50 Å), pi-cation (4.17 Å), Leu56 pi-

			alkyl (5.36 Å), Asn57 hydrogen (2.62 Å), halogen (3.47 Å), Ile61 hydrogen (2.42 Å), Ala77 halogen (3.24 Å), pi-alkyl (5.17 Å), Ala80 pi-alkyl (5.04 Å), Ile85 pi-sigma (3.81 Å), Pro86 alkyl (4.65 Å), pi-alkyl (5.35 Å)
	AJ-030/12105289 (CID: 46215462)	-9.2	Ala87 alkyl (4.69, 5.04 Å), Leu89 alkyl (4.97 Å), pi-alkyl (5.99 Å), pi-sigma (3.71 Å), Ile91 alkyl (4.46 Å), pi-alkyl (4.57, 4.88 Å), Pro92 hydrogen (3.74 Å), pi-alkyl (5.38, 5.78 Å), Asp103 hydrogen (3.22 Å), Ile105 alkyl (4.30 Å), pi-sigma (3.44 Å)
	3-[[4-imidazol-1-yl-6-(4-oxidanylpiperidin-1-yl)-1,3,5-triazin-2-yl]amino]-4-methyl-~{N}-[3-(trifluoromethyl)phenyl]benzamide (CID: 145722473)	-9.2	Asn29 halogen (3.62 Å), His33 halogen (3.17 Å), Pro34 halogen (3.63 Å), Asn35 halogen (3.17 Å), Leu37 pi-alkyl (5.01 Å), Thr51 hydrogen (2.58 Å), Phe55 pi-alkyl (5.75 Å), Arg58 pi-alkyl (4.91 Å), Ile61 alkyl (5.26 Å)
	GSK-1521498 (CID: 24737629)	-9.1	Leu7 pi-alkyl (5.77 Å), His33 hydrogen (2.34 Å), Pro34 alkyl (5.61 Å), pi-sigma (3.97 Å), Asn35 hydrogen (3.09 Å), Phe36 halogen (3.15 Å), Leu37 alkyl (5.21 Å), pi-alkyl (5.38 Å), Val38 pi-alkyl (4.96 Å), Arg58 pi-alkyl (4.96, 5.49 Å), Ile61 pi-alkyl (5.64 Å)
	Nilotinib (CID: 644241)	-9.0	Leu28 pi-alkyl (5.50 Å), Asn29 halogen (3.76 Å), His33 halogen (3.45 Å), Pro34 halogen (2.98 Å), pi-alkyl (3.83, 4.92, 5.28 Å), Asn35 halogen (3.95 Å), Phe36 pi-pi t-shared (5.43 Å), Leu37 pi-alkyl (5.74 Å), Thr51 hydrogen (2.54 Å), Gln54 hydrogen (3.34 Å), Phe55 pi-alkyl (5.36 Å), pi-pi t-shaped (5.59 Å), Arg58 pi-alkyl (5.25, 5.52 Å), Ile61 alkyl (5.63 Å)
	Gemcitabine (CID: 60750)	-7.1	Asn29 hydrogen (2.84 Å), His33 hydrogen (2.59 Å), Pro34 pi-alkyl (5.21 Å), Asn35 hydrogen (2.70 Å), Thr51 hydrogen (2.06 Å), Gln54 hydrogen (2.41 Å), halogen (3.10 Å), Phe55 pi-pi stacked (5.11 Å)
EIF2A	Omilancor (CID: 121299620)	-11.5	Ile124 pi-alkyl (4.90, 5.28), Gln125 hydrogen (2.55), Lys126 pi-alkyl (4.96), Lys127 pi-alkyl (5.39), Arg144 pi-alkyl (4.58), pi-cation (4.92), Asn145 hydrogen (2.40), Val146 pi-alkyl (4.91,

			5.15), Asn147 hydrogen (2.03), Asn148 hydrogen (2.59)
	Atogepant (CID: 72163100)	-11.4	Ile43 pi-sigma (3.57), Lys46 hydrogen (2.33), pi-alkyl (4.43), Glu82 pi-anion (4.24), Pro85 pi-alkyl (4.74), alkyl (4.81), Leu175 pi-alkyl (5.33), Ser412 hydrogen (2.44), Trp413 fluorine (3.25, 3.26), Pro415 alkyl (4.10), fluorine (3.64)
	Vanzacaftor (CID: 139399801)	-11.3	Val174 alkyl (4.12, 4.24), Leu227 pi-alkyl (5.09, 5.11), Lys230 alkyl (4.40, 4.87), Trp281 hydrogen (2.92), pi-alkyl (5.12), Tyr321 hydrogen (3.79), Tyr322 hydrogen (2.24), Trp365 pi-pi T-shaped (5.49)
	BMS-986142 (CID: 86582336)	-11.1	Lys230 pi-alkyl (5.49), Pro324 alkyl (4.12, 4.38), pi-alkyl (4.86), Phe363 hydrogen (2.56), Trp365 hydrogen (1.81, 3.09), Pro367 alkyl (4.21, 4.33), Pro415 alkyl (5.03), Asp418 hydrogen (2.82)
	Tapotoclax (CID: 118910268)	-11.1	Pro85 hydrogen (3.38), pi-alkyl (5.06), alkyl (3.83) Trp135 pi-alkyl (4.99), Pro177 pi-alkyl (4.80), Lys230 hydrogen (2.84), Asp418 hydrogen (3.48)
	Gemcitabine (CID: 60750)	-6.3	Gln125 hydrogen (2.16 Å), Gln129 hydrogen (2.02, 2.93 Å), Asn145 halogen (3.09, 3.41 Å), Asn147 hydrogen (2.90 Å), Asn148 hydrogen (1.93, 2.90 Å)
GSN	Dihydroergotamine (CID: 10531)	-11.4	Asp264 hydrogen (1.78 Å), Arg268 pi-cation;pi-donor (3.96 Å), His309 pi-alkyl (5.71 Å), Gln349 hydrogen (2.00 Å), Leu361 hydrogen (3.75 Å), pi-alkyl (5.09 Å), Gln364 hydrogen (2.34 Å), Asp707 pi-anion (4.69, 4.76 Å), Pro708 alkyl (4.99 Å), pi-alkyl (4.44, 5.32 Å)
	Vevorisertib (CID: 71138858)	-11.2	Gly124 hydrogen (3.15 Å), Phe125 pi-alkyl (4.40 Å), Glu126 hydrogen (3.26 Å), Ala128 pi-alkyl (5.20 Å), Arg172 hydrogen (2.30 Å), Asp264 pi-anion (4.41, 4.55 Å), Arg268 hydrogen (2.80, 2.90 Å), Asp312 pi-anion (3.89 Å), Lys314 pi-alkyl (5.17 Å), Pro345 alkyl (5.10 Å), Leu361 alkyl (5.45 Å), Gln364 hydrogen (3.25 Å), Tyr703 hydrogen (2.53 Å)
	Cirtuvivint (CID: 132056570)	-11.1	Asp264 hydrogen (3.79 Å), pi-anion (4.54 Å), His309 hydrogen (3.68 Å), pi-

			cation (4.69 Å), Asp312 pi-anion (4.82 Å), Gly313 hydrogen (3.56 Å), Lys314 pi-alkyl (5.33, 5.69 Å), Gln349 hydrogen (2.91 Å), Leu361 pi-alkyl (4.53 Å), pi-sigma (3.93 Å), Gln364 hydrogen (2.96 Å), Arg641 alkyl (4.36 Å), pi-alkyl (4.99 Å), Glu655 hydrogen (3.42 Å), Asp675 hydrogen (3.75 Å), Trp677 pi-alkyl (5.40 Å), Tyr703 hydrogen (2.57 Å), Asp707 pi-anion (3.90 Å), Pro708 pi-alkyl (5.08 Å)
	Lifirafenib (CID: 89670174)	-11.1	Phe125 pi-pi t-shaped (5.36 Å), Asp264 hydrogen (3.94 Å), pi-anion (4.70 Å), His309 pi-cation (4.87 Å), Asp312 pi-anion (4.56 Å), Lys314 pi-alkyl (5.45, 5.92 Å), Gln349 hydrogen (3.19 Å), Leu361 alkyl (4.98 Å), halogen (3.39, 3.39 Å), pi-alkyl (5.36 Å), pi-sigma (3.76 Å), Gln364 halogen (3.07, 3.98 Å), Phe365 pi-alkyl (5.08 Å), Glu656 hydrogen (3.06 Å), Asp707 halogen (3.60 Å), Pro708 alkyl (4.36 Å), pi-alkyl (5.78 Å)
	MK-3207 (CID: 25019940)	-11.1	Ala128 pi-alkyl (5.69 Å), Val170 alkyl (5.10 Å), Asp312 amide-pi stacked (5.09 Å), Gly313 amide-pi stacked (5.09 Å), Lys314 pi-alkyl (5.00 Å), Pro345 pi-alkyl (5.91 Å), Gln347 halogen (3.15 Å), Gln349 hydrogen (2.86 Å), Leu361 alkyl (5.49 Å), pi-alkyl (5.25 Å), Gln364 hydrogen (3.60 Å), Pro708 pi-alkyl (4.33 Å)
	Gemcitabine (CID: 60750)	-7.0	Lys363 pi-alkyl (5.62 Å), halogen (3.08 Å), Gln364 halogen (3.14 Å), Phe366 hydrogen (2.13 Å), halogen (2.83, 3.18 Å), Trp369 hydrogen (2.45 Å), Leu630 pi-alkyl (5.76 Å), Ala637 pi-alkyl (4.55 Å), His638 pi-pi T-shaped (4.69 Å), pi-catiom (3.93 Å)
HPCAL1	Milbemycin A3 (CID: 9828343)	-10.6	Tyr52 hydrogen (2.55 Å), pi-alkyl (4.94 Å), Phe55 pi-alkyl (5.94 Å), pi-sigma (3.78 Å), Phe56 pi-alkyl (4.83, 5.13 Å), Phe72 pi-alkyl (5.89 Å), Phe85 pi-alkyl (4.88 Å), Leu89 alkyl (4.20, 4.57, 5.38 Å), Trp103 pi-alkyl (4.98 Å), Ala104 alkyl (4.28 Å), Met107 alkyl (4.62 Å), Gln184 hydrogen (3.72 Å), Pro187 alkyl (3.98 Å)
	Enzastaurin (CID: 176167)	-10.6	Phe64 pi-pi t-shaped (4.98 Å), Lys100 pi-alkyl (5.34 Å), Trp103 pi-alkyl (5.45

			Å), Ile128 pi-alkyl (5.88 Å), Leu183 hydrogen (2.94 Å), Cys185 hydrogen (2.17 Å), Pro187 alkyl (4.92 Å)
	Tirilazad (CID: 104903)	-10.5	Trp30 pi-alkyl (5.33 Å), Phe48 pi-alkyl (4.35 Å), Ile51 alkyl (4.43, 4.88, 5.90 Å), Tyr52 pi-alkyl (5.38 Å), Phe55 pi-alkyl (4.91, 5.07 Å), Phe64 pi-alkyl (4.76 Å), Phe85 pi-alkyl (5.37, 5.94 Å), Leu89 alkyl (4.92 Å), Thr92 hydrogen (3.54 Å), Lys100 alkyl (4.85 Å), Trp103 pi-alkyl (4.91 Å), Ala104 alkyl (4.60 Å), Cys185 alkyl (5.93 Å)
	BMS-955176 (CID: 60152109)	-10.4	Phe34 hydrogen (3.43 Å), Phe48 pi-alkyl (4.74 Å), Ile51 alkyl (4.20, 4.98 Å), pi-alkyl (5.15 Å), Tyr52 pi-alkyl (5.36, 5.95 Å), pi-sigma (3.89 Å), Phe55 pi-alkyl (4.47, 4.73, 4.89, 5.42, 5.76 Å), Phe56 pi-alkyl (4.93, 5.13 Å), Phe85 pi-alkyl (4.54, 5.59 Å), Leu89 alkyl (4.90 Å), Pro187 alkyl (5.12 Å)
	MK-1084 (CID: 156295589)	-10.4	Tyr52 pi-alkyl (5.05 Å), Phe55 pi-alkyl (5.79 Å), pi-pi t-shaped (4.92 Å), Phe64 pi-alkyl (4.72 Å), Trp103 pi-alkyl (4.92 Å)
	Gemcitabine (CID: 60750)	-6.6	Met138 hydrogen (2.64 Å), Glu142 hydrogen (2.98 Å), Arg181 hydrogen (2.43 Å), pi-alkyl (4.04 Å), Leu182 pi-alkyl (5.39 Å), Cys185 hydrogen (3.46 Å), Ser189 halogen (3.89 Å)
MVB12A	Hypericin (CID: 3663)	-7.4	Met215 hydrogen (2.58 Å), Phe220 pi-alkyl (4.74 Å), pi-pi stacked (3.76, 3.93, 4.25, 4.39, 4.82, 4.88, 5.11 Å)
	Apto-253 (CID: 11960271)	-7.2	Ile212 pi-alkyl (5.61 Å), Met215 pi-alkyl (5.42 Å), Asp216 hydrogen (1.97, 2.55 Å), Pro219 pi-alkyl (4.13 Å), pi-sigma (3.95 Å), Phe220 hydrogen (3.11, 3.18, 3.25 Å)
	Tirilazad (CID: 104903)	-7.1	Phe220 pi-alkyl (4.12, 4.88 Å), Leu222 alkyl (4.54 Å), pi-alkyl (5.87 Å), His223 pi-alkyl (5.78 Å)
	Cepharanthine (CID: 10206)	-7.0	Ile212 hydrogen (3.80 Å), alkyl (5.13 Å), pi-alkyl (4.99 Å), Asp216 hydrogen (3.42 Å), Phe220 hydrogen (3.55 Å), pi-alkyl (4.70, 5.73 Å), pi-pi stacked (4.89 Å), pi-pi t-shaped (5.17 Å), Leu222 alkyl (4.30 Å)
	Lestaurtinib (CID: 126565)	-6.9	Phe220 hydrogen (3.13 Å), pi-pi stacked (4.03, 4.07, 4.45, 5.86 Å)
	Gemcitabine (CID: 60750)	-4.2	Ile212 pi-alkyl (5.14 Å), halogen (3.27 Å), Met215 hydrogen (2.81, 3.56 Å), pi-

			alkyl (5.28 Å), Asp216 hydrogen (2.27, 2.43 Å), halogen (3.32, 3.92 Å)
MYH14	Casopitant (CID: 9917021)	-10.9	Asp109 pi-anion (4.37 Å), Ala111 pi-alkyl (5.17 Å), Glu112 pi-anion (3.80 Å), Arg167 hydrogen (2.95, 3.05, 3.27 Å), Leu513 alkyl (4.30, 4.55 Å), pi-alkyl (4.77 Å), halogen (3.33 Å), Arg520 pi-cation (4.85 Å), Arg735 hydrogen (2 .97 Å), halogen (3.71 Å), pi-alkyl (5.22 Å), Glu740 hydrogen (3.86 Å), Lys784 hydrogen (2.67 Å)
	GLPG-1205 (CID: 71616860)	-10.7	Thr283 hydrogen (3.26 Å), Lys288 pi-alkyl (5.19 Å), Glu449 pi-anion (3.15 Å), Arg453 alkyl (4.30 Å), Val456 alkyl (5.81 Å), Phe490 pi-alkyl (5.52 Å), Cys494 alkyl (4.53 Å), Val670 alkyl (4.21 Å), Leu673 alkyl (4.56 Å), pi-alkyl (5.09 Å), amide-pi stacked (4.15 Å), Tyr674 amide-pi stacked (4.15 Å), pi-alkyl (4.78 Å), Arg680 hydrogen (2.39, 2.92 Å)
	HRS-4642 (CID: 171853615)	-10.2	Ala111 alkyl (4.74, 4.85 Å), Gly139 hydrogen (3.72 Å), Lys166 alkyl (5.72 Å), Val177 halogen (2.95 Å), Gly180 hydrogen (3.14 Å), Met510 alkyl (5.77 Å), Leu513 pi-alkyl (4.71, 5.25, 5.62 Å), Asn689 hydrogen (2.30 Å), Pro733 halogen (3.49 Å), Arg735 hydrogen (2.12, 3.80 Å), halogen (3.98 Å)
	Enoxolone (CID: 10114)	-10.1	Ala111 alkyl (3.68, 5.59 Å), His168 pi-alkyl (5.54 Å), pi-sigma (3.68 Å), Met510 alkyl (5.61 Å), Leu513 alkyl (4.41, 5.35 Å), Arg735 alkyl (5.41 Å), Ile736 alkyl (4.82 Å)
	Bemcentinib (CID: 46215462)	-10.0	Arg167 pi-alkyl (5.16 Å), Val177 pi-alkyl (5.73 Å), Gly180 amide-pi stacked (4.56 Å), Ala181 amide-pi stacked (4.56 Å), Ser184 hydrogen (3.49 Å), Asp188 hydrogen (2.92 Å), His508 pi-alkyl (5.73 Å), Val512 alkyl (5.14 Å), pi-alkyl (5.68 Å), Asn689 hydrogen (2.52 Å)
	Gemcitabine (CID: 60750)	-6.9	Ser200 hydrogen (3.63 Å), Thr205 hydrogen (3.56 Å), Glu206 pi-anion (3.69 Å), Glu246 halogen (3.30 Å), pi-anion (4.08 Å), Asn250 hydrogen (3.06 Å), halogen (3.04, 3.28 Å), Asn258 hydrogen (2.63, 2.80 Å), halogen (3.19, 3.97, 3.98 Å)

MYO1C	MK-4232 C-11 (CID: 155907712)	-12.2	Pro47 alkyl (4.99 Å), pi-alkyl (5.76 Å), Phe74 pi-alkyl (5.74 Å), Ala91 alkyl (3.83 Å), pi-alkyl (5.63 Å), Glu95 halogen (3.70 Å), pi-anion (4.37 Å), Leu417 pi-alkyl (5.02 Å), Pro585 alkyl (4.78 Å), pi-alkyl (5.08 Å), Ala628 alkyl (4.62 Å), Pro685 alkyl (5.00 Å), Phe689 pi-alkyl (5.71 Å)
	Brigimadlin (CID: 129264140)	-11.7	Pro47 pi-alkyl (5.56 Å), Ala91 alkyl (4.30 Å), pi-alkyl (4.89 Å), Leu417 hydrogen (2.68 Å), Pro585 pi-alkyl (5.63 Å), Ala628 hydrogen (3.73 Å), alkyl (3.44 Å), halogen (4.57 Å), pi-alkyl (4.57 Å), Pro685 alkyl (3.99 Å), pi-alkyl (5.20 Å), Leu688 alkyl (4.21, 4.97 Å), pi-alkyl (5.42 Å)
	Conivaptan (CID: 151171)	-11.5	Pro47 pi-alkyl (5.88 Å), Arg90 pi-alkyl (5.43 Å), Ala91 pi-alkyl (5.37 Å), pi-sigma (3.79 Å), Pro585 alkyl (5.47 Å), Tyr587 pi-alkyl (5.35 Å), Arg630 hydrogen (3.49 Å), Phe689 pi-pi t-shaped (4.86 Å)
	Atogepant (CID: 72163100)	-11.3	Pro47 alkyl (5.24 Å), halogen (2.86 Å), Thr87 halogen (3.52 Å), Arg90 hydrogen (2.10 Å), Ala91 alkyl (3.58 Å), pi-sigma (3.58 Å), Ala586 alkyl (4.09 Å), Tyr587 hydrogen (2.46 Å)
	Vanzacaftor (CID: 139399801)	-11.2	Pro47 pi-alkyl (5.24 Å), Ala91 alkyl (4.65, 4.71 Å), Leu417 alkyl (5.46 Å), Tyr587 pi-alkyl (5.82 Å), Glu584 hydrogen (2.28 Å), pi-anion (4.68 Å), Tyr629 pi-alkyl (5.84 Å), Leu688 alkyl (5.10 Å), Phe689 pi-alkyl (4.81, 5.01 Å)
	Gemcitabine (CID: 60750)	-6.7	Asp15 pi-anion (4.82 Å), Gly46 hydrogen (3.22 Å), Pro47 hydrogen (3.50 Å), Arg623 hydrogen (1.84, 2.32, 2.77 Å), Gly626 hydrogen (2.91, 3.17 Å), Pro685 hydrogen (3.33 Å), pi-alkyl (4.43 Å)
OTUB1	Bemcentinib (CID: 46215462)	-9.4	Glu60 hydrogen (3.58 Å), Tyr61 pi-alkyl (5.08 Å), Tyr68 pi-alkyl (5.84 Å), Pro87 hydrogen (2.28 Å), Lys213 pi-alkyl (4.88 Å), Pro263 amide-pi stacked (3.98 Å), alkyl (4.98 Å), pi-alkyl (5.43 Å), Gly264 hydrogen (3.10 Å), amide-pi stacked (3.98 Å)
	1144072-40-1 (CID: 45488079)	-9.3	Ser215 halogen (3.34 Å), Asp216 halogen (3.41 Å), His217 hydrogen (2.72, 3.02 Å), pi-pi stacked (4.37 Å), Ile221 pi-alkyl (4.26 Å), Gln225

			hydrogen (2.95 Å), Tyr235 hydrogen (2.76 Å), pi-alkyl (5.15, 5.57 Å), pi-pi stacked (4.41, 4.50 Å), Asn245 hydrogen (1.82 Å), His247 hydrogen (2.46, 3.73 Å), pi-alkyl (4.80 Å), Pro250 alkyl (5.58 Å), Tyr261 alkyl (4.14 Å), Tyr266 hydrogen (2.86 Å)
	15-PGDH-IN-1 (CID: 135300446)	-9.2	Tyr182 pi-alkyl (5.91 Å), Phe189 pi-sigma (3.62 Å), Phe190 pi-alkyl (5.51 Å), His217 pi-sigma (3.92 Å), pi-pi stacked (4.58 Å), Ile221 pi-alkyl (4.52, 5.55 Å), Pro250 pi-alkyl (5.89 Å), Glu251 hydrogen (2.22 Å)
	Tapotoclax (CID: 118910268)	-8.8	Phe203 pi-alkyl (5.63 Å), Cys212 hydrogen (3.58 Å), Lys213 pi-alkyl (5.47 Å), Asp216 pi-anion (3.14 Å), His217 hydrogen (3.71 Å), pi-alkyl (4.82 Å)
	Ubrogepant (CID: 68748835)	-8.7	Lys213 pi-alkyl (4.77 Å), His217 hydrogen (2.11, 2.13 Å), pi-alkyl (5.62 Å), Tyr235 pi-alkyl (6.00 Å), pi-pi stacked (4.72 Å), His247 hydrogen (2.43 Å), Tyr261 hydrogen (2.94 Å), pi-alkyl (5.88 Å), Gly264 hydrogen (2.73, 3.02, 3.32 Å)
	Gemcitabine (CID: 60750)	-6.2	Lys256 halogen (3.05 Å), Leu269 halogen (3.46 Å), Tyr270 hydrogen (3.36 Å), pi-pi T-shaped (4.84 Å), Lys271 hydrogen (2.22, 2.94 Å)
PACSIN2	HRS-4642 (CID: 171853615)	-10.1	Glu130 hydrogen (2.92 Å), Glu133 pi-anion (4.89 Å), Ala134 pi-alkyl (5.36 Å), Gly137 hydrogen (3.42 Å), Lys140 alkyl (4.86, 5.09 Å), Ala141 alkyl (4.25 Å), Gln228 hydrogen (2.62 Å), halogen (3.75 Å), Val229 hydrogen (3.71 Å), halogen (3.92 Å), pi-alkyl (5.06, 5.58 Å), Gln232 pi-sigma (3.83 Å), Cys233 hydrogen (3.34 Å), pi-alkyl (5.29 Å)
	MRTX1133 (CID: 156124857)	-9.85	Glu133 pi-anion (4.91 Å), Ala134 pi-alkyl (5.43 Å), Gly137 hydrogen (3.78 Å), Lys140 alkyl (5.15, 5.68 Å), Ala141 alkyl (4.84 Å), Gln228 halogen (3.40 Å), Val229 hydrogen (3.53 Å), halogen (3.96 Å), pi-alkyl (4.90, 5.28 Å), Gln232 pi-sigma (3.76 Å), Cys233 pi-alkyl (5.44 Å)
	2247950-42-9 (CID: 140944699)	-9.6	Glu133 hydrogen (3.42 Å), pi-anion (4.23, 4.64 Å), Ala134 pi-alkyl (4.77 Å), alkyl (5.50 Å), Gly137 halogen (3.55 Å), Lys140 halogen (3.53 Å),

			Ala141 pi-alkyl (5.66 Å), Val229 alkyl (4.67 Å), pi-alkyl (5.89 Å), Cys233 alkyl (5.94 Å), pi-alkyl (5.25 Å)
	Zavegepant (CID: 53472683)	-9.0	Glu130 hydrogen (3.53, 3.89 Å), Glu133 hydrogen (3.75, 3.76 Å), Ala134 alkyl (5.15 Å), Asp136 hydrogen (2.27 Å), Lys140 alkyl (4.73 Å), pi-alkyl (5.84 Å), Ala141 alkyl (4.97 Å), Pro144 pi-alkyl (4.01, 5.43 Å), Asn225 hydrogen (2.70 Å), Val229 alkyl (5.31 Å)
	Nilotinib (CID: 644241)	-9.0	Ile31 alkyl (4.79 Å), Glu130 hydrogen (3.10 Å), Glu133 hydrogen (2.28, 2.62 Å), amide-pi stacked (4.40 Å), Ala134 alkyl (4.45 Å), pi-alkyl (4.52 Å), amide-pi stacked (4.40 Å), Gly137 hydrogen (3.63 Å), Lys140 hydrogen (2.50, 2.87 Å), pi-alkyl (5.67 Å), Ala141 alkyl (4.03 Å), Val229 pi-alkyl (5.73, 5.91 Å), Cys233 alkyl (4.25 Å), pi-sulfur (4.83 Å), Gln235 hydrogen (3.91 Å), Phe236 pi-alkyl (4.83 Å), pi-pi stacked (4.63 Å), Lys239 hydrogen (2.44 Å)
	Gemcitabine (CID: 60750)	-5.7	Ala85 pi-alkyl (4.90 Å), Ser88 hydrogen (2.19 Å), Ile266 hydrogen (2.65 Å), Ser273 (2.05 Å)
PCCA	1144072-40-1 (CID: 45488079)	-10.1	Arg71 hydrogen (2.75, 3.03, 3.06, 3.81 Å), halogen (3.80 Å), pi-alkyl (5.54 Å) Gly72 hydrogen (3.51 Å), Glu73 hydrogen (2.54 Å), Tyr143 pi-pi t-shaped (5.44 Å), Phe145 pi-alkyl (4.67 Å), pi-pi t-shaped (5.62 Å), His269 hydrogen (3.14 Å), pi-alkyl (4.70 Å), Lys298 pi-alkyl (4.95 Å), pi-cation (3.94 Å), pi-sigma (3.43 Å), Glu336 hydrogen (3.47 Å), Leu348 alkyl (5.65 Å), Val356 pi-alkyl (5.15 Å), Asp446 halogen (3.31 Å), pi-anion (4.69 Å), Met448 alkyl (4,72 Å)
	Methylcholanthrene (CID: 1674)	-10.1	Arg71 pi-alkyl (5.19 Å), Phe145 pi-alkyl (3.92 Å), pi-pi stacked (4.11, 4.75, 4.75 Å), Met448 pi-alkyl (5.80 Å)
	Boditrectinib (CID: 121395201)	-10.1	Arg71 alkyl (5.10 Å), Tyr143 pi-pi t-shaped (5.03, 5.54 Å), halogen (3.29 Å), Gly144 hydrogen (2.97 Å), Phe145 hydrogen (3.03 Å), pi-alkyl (5.50 Å), pi-pi stacked (3.91 Å), Glu148 hydrogen (2.59 Å), Val356 pi-alkyl (5.04, 5.22 Å), Tyr445 hydrogen (3.24

			Å), Asp446 hydrogen (2.49 Å), pi-anion (4.19 Å), Met448 pi-sulfur (5.75 Å)
	PF-07284892 (CID: 155164064)	-9.9	Arg71 pi-alkyl (5.68 Å), Tyr143 pi-alkyl (5.98 Å), Asn296 hydrogen (2.12 Å), Lys298 pi-alkyl (5.48 Å), pi-cation (4.24 Å), Glu349 hydrogen (3.94 Å), Arg353 hydrogen (2.23, 2.97 Å), Val356 pi-sigma (3.65 Å)
	Tirilazad (CID: 104903)	-9.9	Phe145 pi-alkyl (4.81, 5.17, 5.83 Å), Pro412 alkyl (4.78 Å), Ile443 alkyl (5.26 Å), Tyr444 hydrogen (3.22, 3.36 Å), Ala680 alkyl (4.95 Å)
	Gemcitabine (CID: 60750)	-7.1	Arg71 hydrogen (3.07 Å), Gly72 pi-sigma (3.65 Å), Glu73 hydrogen (2.04 Å), Ile74 hydrogen (2.74 Å), Tyr143 halogen (3.48 Å), Met448 hydrogen (2.10 Å), pi-alkyl (4.87 Å)
PCK2	HRS-4642 (CID: 171853615)	-10.9	Gly307 hydrogen (3.17 Å), Asn310 halogen (3.48 Å), Arg454 hydrogen (1.94, 2.42 Å), halogen (3.80 Å), Ser482 halogen (3.30 Å), Phe535 pi-alkyl (5.42 Å), pi-pi t-shaped (4.72, 5.17 Å), Phe543 pi-pi t-shaped (4.97 Å), Phe548 pi-alkyl (5.31 Å), pi-pi stacked (4.09, 4.12 Å)
	Bosmolisib (CID: 134427646)	-10.6	Ala305 pi-alkyl (4.40, 4.88, 5.50 Å), Gly307 hydrogen (3.68 Å), Val353 pi-alkyl (5.35, 5.74 Å), Arg454 hydrogen (2.90, 3.00 Å), pi-cation;pi-donor (3.98 Å), Trp534 pi-alkyl (5.10 Å), Phe535 pi-alkyl (5.26 Å), Phe543 pi-pi t-shaped (5.50 Å), Phe548 pi-alkyl (4.44 Å), pi-pi stacked (4.27, 4.89 Å)
	Dihydroergotamine (CID: 10531)	-10.4	Asn310 hydrogen (3.56 Å), Arg454 hydrogen (3.24 Å), Trp534 pi-pi stacked (5.39 Å), Phe535 pi-pi t-shaped (4.60, 5.01 Å), Phe543 pi-pi t-shaped (5.22 Å), Phe548 pi-alkyl (5.10 Å), pi-pi stacked (4.11, 4.43 Å)
	Benzo[a]pyrene (CID: 2336)	-10.4	Arg454 pi-cation (3.85, 3.99, 4.57 Å), Phe543 pi-pi t-shaped (5.03 Å), Phe548 pi-pi stacked (4.19, 4.44, 4.66, 5.05 Å)
	Galicaftor (CID: 121301049)	-10.4	Ala305 alkyl (5.22 Å), pi-alkyl (4.46 Å), Gly307 hydrogen (3.62, 3.80 Å), halogen (3.73 Å), Thr309 hydrogen (3.05 Å), Asn310 halogen (3.84 Å), Val353 hydrogen (2.40 Å), alkyl (4.07 Å), halogen (3.23 Å), pi-alkyl (4.91 Å), Pro420 pi-alkyl (5.59 Å), Arg454 pi-cation (4.27 Å), Ala484 pi-alkyl (5.66

			Å), Trp534 hydrogen (3.27 Å), Phe535 pi-alkyl (5.37 Å), pi-pi t-shaped (4.76 Å), Phe543 pi-alkyl (4.44 Å), Phe548 hydrogen (2.26 Å), pi-pi stacked (3.94 Å), Asn551 hydrogen (2.46, 3.16 Å)
	Gemcitabine (CID: 60750)	-6.2	Gly168 hydrogen (2.27 Å), Ser172 hydrogen (2.83 Å), Arg173 hydrogen (3.52 Å), Ile174 hydrogen (2.91 Å), Ala201 halogen (3.86 Å), Asp206 halogen (3.15 Å)
PDHB	Lifirafenib (CID: 89670174)	-9.9	Ile90 alkyl (4.07 Å), pi-alkyl (5.31 Å), Trp135 pi-alkyl (4.26, 5.03 Å), pi-pi stacked (3.95, 4.25, 4.71, 4.03 Å), His138 pi-alkyl (4.07 Å), pi-cation (4.63 Å), pi-stacked (4.24 Å) hydrogen (2.91 Å), pi-pi t-shaped (5.06 Å) Glu274 pi-anion (3.51 Å) Phe269 pi-sigma (3.69 Å)
	Milbemycin A3 (CID: 9828343)	-9.9	Pro267 alkyl (4.47 Å), Val293 alkyl (3.81 Å), Arg294 hydrogen (2.60, 2.64, 3.59 Å), Phe321 pi-alkyl (4.22 Å), Ala322 alkyl (4.31, 4.66 Å)
	Dihydroergotamine (CID: 10531)	-9.8	His138 pi-alkyl (4.78 Å), Ile241 hydrogen (3.26 Å), Arg242 pi-cation (3.85, 4.30 Å), pi-alkyl (4.68 Å) Met244 pi-alkyl (4.65 Å), Phe269 pi-alkyl (5.35 Å), Ala273 alkyl (3.87 Å), Glu274 pi-anion (4.01, 4.62 Å) Ala277 pi-alkyl (5.04 Å), Arg278 pi-cation;pi-donor (3.91 Å), Glu281 pi-anion (3.91 Å)
	Natamycin (CID: 138403275)	-9.6	Arg294 alkyl (4.29 Å), Ile313 hydrogen (3.05 Å), Asn311 hydrogen (2.40 Å)
	Padnarsertib (CID: 117779453)	-9.6	Ala134 pi-alkyl (4.75 Å), Trp135 pi-alkyl (4.15, 4.21 Å), Ile241 halogen (3.56 Å), His138 pi-cation (4.56 Å), pi-pi stacked (4.48, 4.97 Å), Ala273 pi-alkyl (5.43 Å), Glu274 pi-anion (4.51 Å), hydrogen (3.09 Å), Cys276 pi-alkyl (4.47 Å)
	Gemcitabine (CID: 60750)	-5.6	Thr296 hydrogen (2.09 Å), Asp318 halogen (3.27, 3.50 Å), Ala322 pi-alkyl (4.13 Å)
PLP2	Tirilazad (CID: 104903)	-8.9	Phe85 pi-alkyl (4.95 Å), Leu89 alkyl (4.49 Å), Cys123 alkyl (4.79, 4.65 Å), pi-alkyl (4.47 Å), Gly126 hydrogen (3.52 Å) Phe133 pi-alkyl (3.95, 4.89 Å)
	Bemcentinib (CID: 46215462)	-8.7	His72 pi-alkyl (4.78 Å) pi-cation (3.97 Å), Pro76 hydrogen (2.73 Å), Pro81 pi-alkyl (4.33, 4.78 Å), Arg139 pi-alkyl

			(4.72 Å), His140 pi-cation (3.76, 4.41 Å), pi-pi t-shaped (4.64 Å)
	MK-4232 C-11 (CID: 155907712)	-8.7	Pro76 pi-alkyl (5.11, 5.31 Å), alkyl (4.56 Å), Asn79 hydrogen (2.86 Å), Arg139 alkyl (4.25 Å), His140 hydrogen (2.33, 3.70 Å), pi-alkyl (5.49 Å)
	GRT6010 (CID: 11956289)	-8.5	Ala92 pi-alkyl (4.10, 5.02 Å), Ile93 pi-alkyl (5.13 Å), Leu96 pi-alkyl (5.37 Å), Tyr127 pi-pi t-shaped (5.99 Å)
	HRS-4642 (CID: 171853615)	-8.4	Glu56 hydrogen (2.61 Å), Leu59 pi-alkyl (4.68 Å), Ile62 alkyl (5.43 Å), Phe63 pi-alkyl (5.14 Å), Ile78 alkyl (5.04 Å), Trp80 pi-alkyl (5.32 Å) Arg87 pi-alkyl (5.14 Å), Ile90 pi-alkyl (5.28 Å), pi-sigma (3.70 Å)
	Gemcitabine (CID: 60750)	-5.0	Trp82 hydrogen (2.33 Å), Pro134 hydrogen (2.11 Å), halogen (2.91 Å), Gln137 halogen (2.83, 3.26 Å), Arg139 hydrogen (2.35, 3.28 Å)
POLR2H	Lestaurtinib (CID: 126565)	-11.1	Leu64 alkyl (5.25 Å) pi-alkyl (5.19, 5.21 Å), pi-sigma (3.96 Å), Tyr65 pi-alkyl (4.74 Å), Glu74 hydrogen (2.59 Å), Asn76 hydrogen (1.84, 2.45 Å), Tyr75 pi-pi t-shaped (5.50, 5.02, 5.11 Å), Asp79 hydrogen (3.50 Å), Ala85 pi-alkyl (4.36 Å), pi-sigma (3.80 Å), Val91 hydrogen (2.48 Å), Tyr93 pi-pi t-shaped (4.99 Å), Tyr142 pi-pi t-shaped (5.31, 5.26 Å), pi-donor (2.53, 3.32 Å)
	UCN-01 (CID: 3078519)	-10.9	Leu64 alkyl (4.26 Å), pi-alkyl (5.48, 5.49 Å), pi-sigma (3.91 Å), Tyr65 pi-alkyl (4.66 Å), Glu74 hydrogen (3.61 Å), Tyr75 pi-pi t-shaped (4.71, 4.92 Å) Asn76 hydrogen (2.31 Å), Asp79 hydrogen (3.48 Å), Ala85 pi-alkyl (3.96, 5.13 Å), pi-sigma (3.54 Å), Val91 hydrogen (2.32 Å), pi-alkyl (5.24 Å), Met92 hydrogen (2.98 Å), Tyr142 pi-pi t-shaped (5.43, 5.62 Å), pi-donor (2.93 Å)
	TMC-647055 (CID: 44556044)	-10.7	Tyr65 pi-alkyl (5.36 Å), Glu74 hydrogen (3.63 Å), Tyr75 pi-alkyl (5.11 Å), pi-pi t-shaped (5.29 Å), Arg81 hydrogen (3.26 Å), Ala85 hydrogen (2.72 Å), pi-alkyl (3.85, 4.26 Å), alkyl (5.12 Å), Asp86 hydrogen (3.16, 3.58 Å), Val91 pi-alkyl (4.20, 5.44 Å)
	Zavegepant (CID: 53472683)	-10.6	Glu74 hydrogen (3.44 Å), Tyr75 pi-alkyl (4.93, 5.2 Å), pi-pi stacked (4.48

			Å), Asp79 hydrogen (3.56 Å), Tyr90 hydrogen (2.34 Å), Val91 hydrogen (3.58 Å), Met92 alkyl (4.51 Å), Tyr93 hydrogen (3.20 Å), pi-alkyl (5.48 Å), Leu121 pi-sigma (3.75, 4.00 Å), Tyr142 hydrogen (2.22 Å), pi-alkyl (5.11 Å)
	Tivantinib (CID: 11494412)	-10.2	Leu64 pi-alkyl (5.40 Å), Gly73 hydrogen (3.57 Å), Glu74 hydrogen (3.61 Å), Tyr75 pi-pi t-shaped (4.87, 5.03 Å), pi-alkyl (4.99 Å), Tyr93 pi-alkyl (4.39 Å), Tyr142 pi-pi t-shaped (5.27, 5.37 Å) hydrogen (2.88 Å), pi-donor (2.68 Å)
	Gemcitabine (CID: 60750)	-6.4	Leu64 pi-alkyl (5.39 Å), Asp72 hydrogen (2.54 Å), Tyr75 pi-pi T-shaped (4.90 Å), Asn76 hydrogen (2.18, 3.05 Å), halogen (2.84, 3.45 Å), Asp79 hydrogen (2.28 Å), Tyr142 hydrogen (3.35 Å), pi-pi T-shaped (5.42 Å)
SCO1	Tirilazad (CID: 104903)	-9.2	His168 hydrogen (3.62 Å), Pro170 alkyl (4.21, 4.43, 5.00 Å), Pro174 alkyl (4.87 Å), Ala212 alkyl (5.46 Å), Tyr216 pi-alkyl (5.28 Å), Phe220 pi-alkyl (5.33 Å), Asp255 hydrogen (3.4 Å), Ile257 alkyl (4.35, 5.14 Å)
	Tapotoclax (CID: 118910268)	-9.1	Thr167 hydrogen (3.62 Å), His168 pi-alkyl (5.35 Å), hydrogen (3.59 Å), Pro174 pi-alkyl (4.54 Å), alkyl (4.22 Å), Tyr216 pi-alkyl (5.48 Å), pi-pi t-shaped (5.04 Å), Phe220 pi-alkyl (5.32 Å)
	Padnarsertib (CID: 117779453)	-8.9	Leu135 hydrogen (2.45 Å), pi-cation; pi-donor (2.82 Å), pi-alkyl (4.80, 5.05, 5.49 Å), Leu136 pi-sigma (4.00 Å), pi-alkyl (4.81 Å), pi-donor (3.19 Å), Arg242 hydrogen (3.02 Å), pi-alkyl (4.20, 5.06 Å), Leu274 halogen (2.96 Å), Tyr276 pi-pi stacked (4.18 Å)
	Quarfloxin (CID: 11635763)	-8.8	His168 pi-cation (3.42 Å), pi-pi t-shaped (4.44 Å), Pro170 pi-alkyl (4.84 Å), Ala212 alkyl (5.40 Å), Asn215 halogen (3.30 Å), Tyr216 pi-pi t-shaped (4.99, 5.16, 5.59 Å)
	Atogepant (CID: 72163100)	-8.7	His168 hydrogen (3.51 Å), Cys169 amide-pi stacked (4.75 Å), Pro170 pi-alkyl (3.97 Å), alkyl (4.02 Å), amide-pi stacked (4.75 Å), Pro174 alkyl (4.29 Å), Asn215 halogen (3.20 Å), Tyr216 pi-alkyl (5.39 Å), hydrogen (3.59 Å), Phe220 pi-alkyl (4.76 Å), Asp251

			hydrogen (2.33 Å), Ile257 alkyl (5.48 Å), pi-alkyl (5.30 Å)
	Gemcitabine (CID: 60750)	-5.6	Glu176 hydrogen (2.06 Å), Lys179 pi-alkyl (4.55 Å), pi-cation (4.24 Å), Gln279 hydrogen (2.39 Å), Arg282 halogen (3.66 Å), Lys283 hydrogen (2.21 Å)
SCPEP1	Omilancor (CID: 121299620)	-12.2	Pro32 pi-alkyl (5.48 Å), Gly36 pi-donor (3.05 Å), Thr59 pi-sigma (3.91 Å), Asn60 hydrogen (2.03 Å), amide-pi stacked (3.90 Å), Ser61 hydrogen (2.90 Å), amide-pi stacked (3.90 Å), Pro93 pi-alkyl (4.80, 5.01 Å), Leu94 pi-alkyl (5.30, 5.45 Å), Pro100 pi-alkyl (5.20 Å)
	Zavegepant (CID: 53472683)	-12.1	Phe85 pi-alkyl (4.77 Å), Glu89 hydrogen (1.98 Å), Asn268 hydrogen (2.77, 3.57 Å), Gln298 hydrogen (2.46 Å), Arg299 hydrogen (3.62 Å), Arg302 pi-alkyl (4.72 Å), amide-pi stacked (4.78. 5.33 Å), His303 amide-pi stacked (4.78. 5.33 Å), Asp436 hydrogen (3.23, 3.53 Å)
	Atogepant (CID: 72163100)	-10.5	Met258 alkyl (4.44 Å), Glu261 halogen (3.16, 3.39, 2.88, 3.50 Å), hydrogen (3.47 Å), Asp265 amide-pi stacked (4.78 Å), Gly266 amide-pi stacked (4.78 Å), Thr277 hydrogen (3.06 Å), Cys295 hydrogen (3.64 Å), alkyl (4.26 Å), Gln298 hydrogen (2.25 Å), Arg299 pi-alkyl (5.43 Å), hydrogen (2.2, 2.67 Å), Arg302 hydrogen (3.03 Å)
	ABN401 (CID: 118364782)	-10.4	Glu261 pi-anion (3.51, 3.99 Å), Asn268 pi-donor (3.03 Å), Ser276 hydrogen (3.52, 3.70 Å), Thr277 hydrogen (3.07 Å), Gln298 hydrogen (2.87, 3.78 Å), Arg299 alkyl (5.40 Å), pi-cation (3.45 Å), His303 pi-pi stacked (5.16 Å), Arg306 pi-cation (3.65 Å)
	ITI-214 (CID: 42639643)	-10.3	Asn268 hydrogen (2.52 Å), Tyr270 halogen (3.23 Å), Gln298 amide-pi stacked (4.27 Å), Arg299 pi-alkyl (3.94 Å), amide-pi stacked (4.27 Å), Arg302 alkyl (4.35 Å), Arg306 alkyl (4.77 Å), pi-cation (3.60 Å), Met432 pi-alkyl (4.90 Å), Asp436 halogen (3.66 Å)
	Gemcitabine (CID: 60750)	-7.2	Asn268 hydrogen (2.52, 2.85, 3.08 Å), Ser275 hydrogen (2.93 Å), Thr277 hydrogen (2.18 Å), Gln298 hydrogen (2.13 Å), halogen (3.73 Å), Arg299 hydrogen (2.96 Å), halogen (3.81 Å)

SERPINB6	Quarfloxin (CID: 11635763)	-10.5	Trp173 hydrogen (3.07 Å), Asp174 hydrogen (2.71 Å), Glu175 hydrogen (3.64 Å), Gln176 pi-donor (2.66 Å), Lys179 pi-alkyl (5.28 Å), Val224 alkyl (4.65 Å), Ala330 alkyl (4.58 Å), Ala331 pi-alkyl (5.16, 5.40 Å)
	BMS-986142 (CID: 86582336)	-10.3	Glu175 halogen (3.28 Å), Asp178 hydrogen (3.78 Å), Lys202 pi-alkyl (5.25 Å), Ala331 alkyl (4.25 Å)
	Hypericin (CID: 3663)	-10.2	Glu175 pi-anion (3.84, 3.87, 4.80 Å), hydrogen (2.88 Å), Lys202 alkyl (4.22 Å), Ala330 hydrogen (2.47 Å)
	3-[[4-imidazol-1-yl-6-(4-oxidanylpiperidin-1-yl)-1,3,5-triazin-2-yl]amino]-4-methyl-~{N}-[3-(trifluoromethyl)phenyl]benzamide (CID: 145722473)	-10.2	Gln176 pi-donor (2.67 Å), Phe177 halogen (3.15 Å), Lys179 hydrogen (2.62, 3.50 Å), alkyl (4.02 Å), Phe201 hydrogen (3.07 Å), Gln203 hydrogen (3.74 Å), Glu326 halogen (3.13 Å), Ala331 pi-alkyl (4.17 Å)
	RK-582 (CID: 138594194)	-10.2	Gln176 hydrogen (2.37, 2.65, 3.55 Å), Asp178 hydrogen (3.71 Å), Asn181 hydrogen (2.48, 2.57 Å), Ala331 alkyl (4.57 Å), Ala336 hydrogen (3.58 Å), alkyl (4.18, 4.81 Å), Met338 alkyl (4.12, 4.48, 4.97 Å)
	Gemcitabine (CID: 60750)	-6.6	Asn23 hydrogen (2.19, 2.68 Å), halogen (3.94 Å), Val189 hydrogen (2.41 Å), Asp240 hydrogen (3.19 Å), halogen (2.88 Å), Leu241 hydrogen (2.80, 2.93 Å), Pro354 pi-alkyl (4.42 Å), Ser374 hydrogen (2.70, 3.74 Å)
SFXN2	Padnarsertib (CID: 117779453)	-10.9	Met86 hydrogen (3.53 Å), Asn87 hydrogen (2.65 Å), Gly98 halogen (3.04 Å), Ile101 alkyl (5.40 Å), Ile102 alkyl (5.25 Å), Ala128 pi-alkyl (4.04 Å), Leu129 pi-sigma (3.45, 3.57 Å), pi-alkyl (5.38 Å), Thr133 hydrogen (2.40 Å), pi-sigma (3.65 Å), Tyr150 pi-pi t-shaped (5.09 Å)
	Zavegepant (CID: 53472683)	-10.7	Trp15 pi-alkyl (4.99 Å), Arg14 hydrogen (2.51 Å), Asn29 hydrogen (3.45 Å), Asp32 hydrogen (3.54 Å), Arg34 pi-alkyl (4.85 Å), Trp45 pi-pi t-stacked (3.71, 4.17, 4.50, 5.60 Å), Glu65 hydrogen (3.69 Å), Gln66 hydrogen (1.90 Å), Tyr69 pi-alkyl (4.39, 5.31 Å), pi-pi t-shaped (4.73, 5.10 Å)

	Laniquidar (CID: 6450806)	-10.2	His79 pi-pi stacked (4.11, 5.75 Å), pi-cation (4.11 Å), Trp121 hydrogen (3.06 Å), Ser125 hydrogen (2.65 Å), Ala128 alkyl (4.47 Å), pi-sigma (3.72 Å), Leu129 alkyl (5.10 Å), pi-alkyl (5.25 Å), Thr132 pi-alkyl (4.60 Å), pi-pi t-shaped (5.21 Å), Thr133 hydrogen (3.46, 3.47 Å), Ala137 pi-alkyl (5.34 Å), Tyr150 pi-alkyl (4.82 Å), pi-pi t-shaped (5.19 Å)
	SYHA1813 (CID: 118331338)	-10.1	Ile89 pi-alkyl (5.03, 5.09 Å), Ile102 pi-alkyl (5.37 Å), pi-sigma (3.90 Å), Val122 pi-alkyl (4.75 Å), Ser125 hydrogen (3.27 Å), Ala128 pi-alkyl (4.02 Å), alkyl (3.95 Å), Leu129 pi-alkyl (5.35, 5.47 Å), pi-sigma (3.54 Å), Tyr132 pi-alkyl (5.08 Å), Tyr150 pi-pi t-shaped (5.06 Å)
	JNJ-49095397 (RV568) (CID: 45109868)	-10.1	Pro13 alkyl (4.79 Å), pi-alkyl (5.42 Å), Arg18 pi-cation (4.26 Å), pi-alkyl (4.72 Å), pi-sigma (3.53 Å), Lys207 alkyl (4.79 Å), Gln227 hydrogen (2.44 Å), Pro287 pi-alkyl (4.46 Å), Lys289 hydrogen (1.96 Å), pi-alkyl (3.80, 4.51, 4.56 Å), alkyl (3.95 Å), Glu291 hydrogen (2.08, 3.79 Å), pi-anion (4.15 Å)
	Gemcitabine (CID: 60750)	-6.3	Tyr110 halogen (3.73 Å), Asn165 hydrogen (2.36, 2.65 Å), Thr168 halogen (3.62 Å), Lys169 hydrogen (3.91 Å), Pro173 pi-alkyl (5.33 Å), Gly176 hydrogen (3.13, 3.23 Å)
SH3BGRL3	Rebastinib (CID: 25066467)	-9.2	Ala59 alkyl (4.22 Å), Tyr70 halogen (3.44 Å), Cys71 hydrogen (2.19 Å), halogen (3.06 Å), Asp73 hydrogen (2.26 Å), Leu76 pi-alkyl (5.05 Å), pi-sigma (3.63 Å), Ala80 pi-alkyl (5.1 Å), pi-sigma (3.96 Å), Glu88 amide-pi stacked (3.75, 4.65 Å), Phe89 amide-pi stacked (3.75, 4.65 Å), Lys91 pi-alkyl (5.36 Å)
	Padnarsertib (CID: 117779453)	-9.1	Ser2 hydrogen (2.64 Å), Leu4 hydrogen (2.37, 3.54 Å), Thr9 halogen (3.14 Å), Ser10 hydrogen (3.34 Å), Asp29 pi-anion (3.71 Å), Ile33 halogen (3.69 Å), Tyr35 hydrogen (2.90 Å), pi-pi stacked (4.55 Å), Leu37 pi-alkyl (4.92 Å), pi-sigma (3.61 Å)
	Bemcentinib (CID: 46215462)	-9.1	Tyr70 pi-pi t-shaped (5.42, 5.66 Å) pi-sigma (3.57 Å), Leu76 pi-alkyl (5.31

			Å), Ala80 pi-alkyl (4.84 Å), Glu88 amide-pi stacked (4.51 Å), Phe89 amide-pi stacked (4.51 Å)
	Zongertinib (CID: 160283094)	-9.1	Ala54 pi-alkyl (4.94 Å), Asn56 pi-donor (2.47 Å), Ala59 pi-alkyl (4.82 Å), Tyr70 pi-alkyl (4.82 Å), Asp73 pi-anion (3.60 Å), Leu76 alkyl (5.14 Å), pi-alkyl (5.14, 5.46 Å), Ala80 alkyl (4.26 Å), Phe89 pi-alkyl (4.97 Å), Lys91 hydrogen (2.44 Å)
	RAF-265 (CID: 11656518)	-9.1	Ala54 pi-alkyl (4.87 Å), Asn56 pi-donor (2.57 Å), Ala59 hydrogen (3.22 Å), pi-alkyl (4.63 Å), Thr60 hydrogen (2.06 Å), Gln63 hydrogen (3.43 Å), Tyr70 pi-pi t-shaped (4.97, 5.51 Å), hydrogen (3.43 Å), Asp73 hydrogen (2.57 Å), halogen (2.96 Å), Leu76 pi-alkyl (5.01 Å), halogen (3.10 Å), Ala80 alkyl (3.94 Å), Thr85 hydrogen (2.72 Å), Phe89 pi-pi t-shaped (4.92 Å), pi-alkyl (5.47 Å)
	Gemcitabine (CID: 60750)	-5.7	Glu16 hydrogen (2.23 Å), Thr60 hydrogen (1.98, 2.43 Å), Pro61 hydrogen (2.14 Å), Pro62 hydrogen (3.70 Å), Gln63 hydrogen (2.72 Å), Asp73 halogen (2.97 Å), Glu75 pi-anion (3.87 Å)
SNAP23	Nilotinib (CID: 644241)	-9.6	Arg67 alkyl (3.74 Å), Glu70 hydrogen (3.14 Å), halogen (3.2, 3.37 Å), Lys71 alkyl (4.76 Å), pi-alkyl (5.34, 5.44 Å), Thr74 pi-sigma (3.49 Å),Val84 alkyl (3.92 Å), pi-sigma (3.80, 3.83 Å), Arg89 pi-alkyl (4.98 Å), alkyl (4.63 Å), Lys91 halogen (2.99, 3.44 Å), Phe93 hydrogen (2.13 Å), Arg204 hydrogen (2.46, 2.54, 2.70 Å)
	RAF-265 (CID: 11656518)	-9.4	Lys64 pi-alkyl (5.42 Å), Arg67 pi-cation;pi-donor (3.34 Å), Thr101 halogen (3.68 Å), Thr102 hydrogen (3.25 Å), Trp103 pi-pi t-shaped (5.32 Å), hydrogen (2.46 Å), halogen (3.01, 3.31 Å), Gly104 halogen (3.64 Å), Asp105 hydrogen (2.26 Å), Ser110 hydrogen (2.19 Å), Cys112 alkyl (5.32 Å), Arg186 hydrogen (3.52 Å), alkyl (4.49 Å), pi-alkyl (4.61 Å), Asp189 halogen (3.02, 3.36 Å)
	3-[[4-imidazol-1-yl-6-(4-oxidanylpiperidin-1-	-9.3	Asn53 hydrogen (3.63 Å), Glu56 pi-anion (3.44, 3.93 Å)Glu57 hydrogen (3.69 Å), Asp60 hydrogen (2.23 Å),

	yl)-1,3,5-triazin-2-yl]amino]-4-methyl-~{N}-[3-(trifluoromethyl)phenyl]benzamide (CID: 145722473)		Thr102 hydrogen (3.45 Å), Ser110 hydrogen (2.88 Å), Cys112 hydrogen (3.59 Å), Arg186 hydrogen (3.22, 3.35 Å), halogen (3.10 Å), alkyl (3.97 Å), pi-alkyl (4.91 Å), Asp189 halogen (2.97, 3.06 Å), Lys190 pi-cation (4.30 Å)
	MK-3207 (CID: 25019940)	-9.3	Ile39 pi-alkyl (5.22 Å), Ile42 pi-sigma (3.97 Å), Asp46 halogen (3.03 Å), Asn165 hydrogen (2.13 Å), Asn172 hydrogen (3.64 Å), Met169 pi-alkyl (5.30 Å), Glu176 halogen (3.50 Å), pi-anion (4.05 Å)
	Bemcentinib (CID: 46215462)	-9.1	Arg12 pi-alkyl (5.09 Å), Ala13 pi-alkyl (4.70, 4.96 Å), Ile16 pi-alkyl (4.48, 5.18 Å), alkyl (4.90 Å), Ile140 alkyl (5.31 Å), pi-sigma (3.53 Å), Ile143 pi-alkyl (4.85 Å)
	Gemcitabine (CID: 60750)	-5.7	Arg67 halogen (3.88 Å), Glu70 halogen (3.18 Å), Thr90 hydrogen (1.89 Å), Asn92 hydrogen (2.65 Å), Arg204 hydrogen (2.12 Å)
SPTAN1	Lomitapide (CID: 9853053)	-10.6	Ile15 pi-alkyl (4.57, 5.40 Å), Arg18 pi-cation (4.08, 4.87 Å), hydrogen (2.36, 2.90 Å), Asp69 halogen (2.98 Å), Glu70 halogen (3.53 Å), Asn71 hydrogen (1.88 Å), halogen (3.07 Å), Lys73 hydrogen (3.01 Å), Asp74 halogen (3.34, 3.53 Å), Leu78 alkyl (4.42 Å), Lys81 alkyl (4.45 Å), hydrogen (2.3, 3.28 Å), Glu140 amide-pi stacked (4.31 Å), Lys141 hydrogen (3.27 Å), alkyl (5.12 Å), pi-alkyl (3.78, 5.18 Å), amide-pi stacked (4.31 Å), Lys144 hydrogen (2.40 Å), pi-alkyl (4.60 Å), Leu145 halogen (3.58 Å)
	Adavivint (CID: 135565709)	-10.4	Leu40 alkyl (4.86 Å), Glu70 hydrogen (3.54 Å), Asp74 pi-anion (3.41, 4.12 Å), Thr76 hydrogen (2.71 Å), pi-donor (3.20 Å), Asn77 hydrogen (2.49, 2.56 Å), Lys81 pi-alkyl (4.95, 5.03 Å), Gln83 hydrogen (2.42 Å), Lys84 hydrogen (2.85 Å), pi-alkyl (4.44, 4.52, 5.01, 5.16 Å) Ala87 pi-alkyl (5.18 Å)
	ONO-7475 (CID: 90645873)	-10.4	Phe48 pi-pi t-shaped (4.99 Å), Glu55 pi-anion (3.69, 4.47 Å), Leu56 pi-alkyl (5.29 Å), Asn95 hydrogen (3.01 Å), Ala98 hydrogen (3.71 Å), pi-alkyl (4.13, 4.14, 4.73 Å), Lys101 alkyl (4.72 Å), Lys101 alkyl (4.88 Å), pi-cation (3.54 Å), Leu102 alkyl (3.79 Å),

			Glu104 pi-anion (4.56 Å), Leu108 alkyl (5.37 Å)
	Omilancor (CID: 121299620)	-10.4	Arg36 pi-alkyl (4.76 Å), Leu40 pi-sigma (3.38, 4.00 Å), Asp74 hydrogen (2.85 Å), pi-anion (4.69 Å), Thr76 pi-donor (2.86 Å), Gly80 hydrogen (2.93 Å), Lys81 pi-alkyl (5.22 Å), Gln83 hydrogen (2.52 Å), Lys84 pi-alkyl (4.88, 5.11, 5.26 Å), Ala87 pi-alkyl (4.87 Å)
	Relacorilant (CID: 73051463)	-10.3	Ile15 pi-alkyl (4.91 Å), Arg18 hydrogen (2.86 Å), Asp74 halogen (2.96, 3.04 Å), amide-pi stacked (5.28 Å), Pro75 halogen (3.11, 3.32 Å), amide-pi stacked (5.28 Å), Thr76 halogen (3.09, 3.23, 3.47 Å), Leu78 pi-alkyl (5.01 Å), alkyl (4.46 Å), hydrogen (2.99 Å), Lys81 alkyl (4.46 Å), hydrogen (2.72 Å), Lys141 alkyl (3.95 Å), Leu145 hydrogen (3.66 Å), Gln147 hydrogen (2.68, 2.77 Å)
	Gemcitabine (CID: 60750)	-7.3	Arg18 hydrogen (2.14, 2.60 Å), Lys73 hydrogen (3.52, 3.67 Å), Asp74 hydrogen (2.39 Å), Thr76 hydrogen (2.55 Å), Leu78 pi-alkyl (5.79 Å), Lys81 pi-alkyl (5.87 Å), Leu145 halogen (3.80 Å), Gln147 hydrogen (2.37 Å), halogen (3.10, 3.73 Å)
STAM	Bemcentinib (CID: 46215462)	-9.6	Ile218 pi-sigma (3.72), Ala233 alkyl (5.30), Thr265 pi-sigma (4.00), Asp267 hydrogen (2.85), Ala270 hydrogen (2.58), pi-alkyl (4.61, 5.02), Met274 alkyl (5.12, 5.18)
	Omilancor (CID: 121299620)	-9.3	Ile218 pi-sigma (3.64), alkyl (5.30), pi-alkyl (5.38), Ala233 pi-alkyl (5.33), Val264 hydrogen (2.51), Ala266 pi-alkyl (5.26), Ala270 pi-alkyl (5.14), Met274 pi-sigma (3.58)
	APTO-253 (CID: 11960271)	-9.2	Arg216 alkyl (4.55), Ile218 alkyl (5.08), pi-alkyl (5.44), Tyr219 pi-pi T-shaped (5.23), Ala233 pi-alkyl (3.89, 4.84), Ala270 pi-alkyl (4.76, 4.97), Pro272 alkyl (5.05), Met274 pi-alkyl (4.09) pi-sigma (3.69), Ile275 pi-alkyl (5.18)
	RK-582 (CID: 138594194)	-9.2	Ile218 pi-alkyl (5.21), alkyl (4.82), fluorine (3.22), Tyr219 hydrogen (2.65), Asp220 hydrogen (2.36), pi-anion (4.87), Ala233 alkyl (4.79), pi-alkyl (5.19), Gly234 hydrogen (2.86), Ala270 alkyl (5.45), Pro272 pi-alkyl (5.13),

			Glu273 hydrogen (3.54), Met274 alkyl (4.84)
	Ocifisertib (CID: 58486178)	-9.1	Arg87 pi-alkyl (5.34), Ser94 hydrogen (2.29), Gln124 hydrogen (2.52), Ser126 hydrogen (2.17), Leu127 pi-alkyl (5.37, 5.46), Met131 alkyl (4.35), Asn134 hydrogen (3.76), Leu135 alkyl (3.99), Asn361 hydrogen (2.51), Val362 pi-alkyl (4.83, 5.02, 5.35)
	Gemcitabine (CID: 60750)	-4.8	Ala223 pi-sigma (3.68 Å), Asp226 hydrogen (2.43 Å), Glu228 hydrogen (3.71 Å), Gln254 halogen (3.22 Å), Ile256 hydrogen (2.44, 2.97 Å)
SURF1	Bemcentinib (CID: 46215462)	-10.0	Pro119 pi-alkyl (4.77 Å), Pro142 alkyl (5.41 Å), pi-alkyl (4.37, 5.30 Å), Met206 pi-sulfur (4.63 Å), Phe250 pi-alkyl (4.86 Å), pi-pi stacked (5.20 Å), Gly261 hydrogen (2.46 Å), Thr263 hydrogen (2.68 Å)
	HRS-4642 (CID: 171853615)	-9.4	Pro70 alkyl (5.1 Å), Val71 alkyl (5.04, 5.14 Å), Phe74 pi-alkyl (5.13, 5.17 Å), Tyr279 pi-pi stacked (3.96, 4.94 Å), halogen (3.29 Å), Ser282 amide-pi stacked (4.16, 4.82 Å), Ala283 pi-alkyl (4.63, 5.13 Å), alkyl (4.01 Å), pi-sigma (3.62 Å), amide-pi stacked (4.16, 4.82 Å)
	MK-3207 (CID: 25019940)	-9.3	Arg144 alkyl (4.59 Å), Glu145 pi-anion (4.04 Å), Glu148 hydrogen (1.96 Å), Leu151 hydrogen (3.63 Å), Ile152 pi-sigma (3.90 Å), Thr156 hydrogen (2.78 Å), Arg264 hydrogen (2.86 Å), Arg268 pi-alkyl (5.18 Å)
	Telcagepant (CID: 11319053)	-9.3	Glu145 hydrogen (3.55 Å), Glu148 hydrogen (2.11 Å), Leu267 halogen (3.42 Å), Arg268 alkyl (5.29 Å), Asn269 hydrogen (2.67 Å)
	Dihydroergotamine (CID: 10531)	-9.2	Glu95 hydrogen (2.84 Å), Val98 pi-alkyl (4.64 Å), Pro102 alkyl (5.44 Å), Pro119 alkyl (4.65 Å), Phe250 hydrogen (2.27 Å), Thr263 hydrogen (3.44, 3.74 Å), Val265 alkyl (4.72 Å), pi-alkyl (4.47 Å), pi-sigma (3.66 Å)
	Gemcitabine (CID: 60750)	-6.3	Met139 hydrogen (2.49 Å), Glu148 pi-anion (4.66 Å), Ser154 hydrogen (2.40 Å), Thr156 hydrogen (2.36 Å)
TIMM50	Cirtuvivint (CID: 132056570)	-9.1	Thr294 hydrogen (3.69 Å), Pro296 alkyl (4.12 Å), pi-alkyl (5.49 Å), Met309 amide-pi stacked (4.31 Å), Glu310 pi-anion (3.53, 4.51 Å), amide-

			pi stacked (4.31 Å), Lys319 hydrogen (3.37 Å), pi-alkyl (3.83 Å), Arg332 pi-alkyl (5.27 Å), Lys349 pi-alkyl (4.85, 5.04 Å), pi-sigma (3.81 Å), Ala352 pi-alkyl (4.40 Å)
	Omilancor (CID: 121299620)	-9.1	Met309 pi-sulfur (5.41, 5.60 Å), Glu310 pi-anion (3.88, 4.51 Å), Glu315 pi-anion (3.91 Å), Lys319 hydrogen (3.05, 3.07 Å), alkyl (5.50 Å), pi-alkyl (4.00, 5.38 Å), Arg322 pi-alkyl (5.39 Å), Lys349 pi-alkyl (4.61 Å), Ala352 pi-alkyl (4.24 Å)
	MK-3207 (CID: 25019940)	-9.0	Glu199 halogen (2.93, 3.31 Å), Asp200 halogen (2.97 Å), Ser239 hydrogen (2.39, 3.08 Å), Tyr241 pi-pi t-shaped (4.72 Å), pi-alkyl (4.72 Å), Met243 pi-alkyl (5.39 Å), Lys265 hydrogen (3.28 Å), pi-alkyl (5.48 Å), Asp293 halogen (2.84 Å), Asp295 hydrogen (2.93, 3.69 Å), Ser298 hydrogen (2.85 Å)
	Risvodetinib (CID: 122650693)	-8.8	Glu173 hydrogen (2.20 Å), pi-anion (3.52 Å), Pro181 pi-alkyl (5.47 Å), Pro182 hydrogen (3.37 Å), Pro184 pi-alkyl (5.29 Å), Arg190 pi-alkyl (5.27 Å), Asn262 hydrogen (3.40, 3.59 Å), His267 pi-alkyl (5.02 Å)
	Bemcentinib (CID: 46215462)	-8.8	Pro184 alkyl (3.66 Å), pi-alkyl (3.68 Å), Arg190 pi-cation (4.11 Å), Ser229 hydrogen (2.32 Å), Ala257 alkyl (5.40 Å), pi-alkyl (4.15 Å)
	Gemcitabine (CID: 60750)	-6.2	Pro184 hydrogen (3.79 Å), Tyr188 halogen (3.08 Å), Arg190 hydrogen (2.68, 2.76 Å), Ser229 hydrogen (2.19 Å), Ser260 hydrogen (2.34 Å), Tyr261 hydrogen (2.56 Å)
VIL1	Bemcentinib (CID: 46215462)	-11.8	Glu105 pi-anion (3.28 Å), Ala106 alkyl (3.78 Å), pi-sigma (3.57 Å), Val341 pi-sigma (3.75 Å), Tyr681 pi-pi t-shaped (5.32 Å), Pro686 alkyl (5.10 Å)
	Padnarsertib (CID: 117779453)	-11.4	Arg108 pi-cation (3.98 Å), hydrogen (3.08 Å), Leu293 pi-alkyl (5.01, 5.34 Å), Lys294 pi-alkyl (5.01 Å), Glu623 hydrogen (2.11 Å), Leu632 pi-alkyl (5.17 Å), Pro686 alkyl (5.35 Å)
	Tirilazad (CID: 104903)	-11.2	Gln113 hydrogen (2.53 Å), Glu196 pi-anion (4.38 Å), Val393 alkyl (4.02, 5.37 Å), Leu442 alkyl (5.00 Å), Ile477 alkyl (5.19 Å), Arg478 hydrogen (3.20 Å), alkyl (4.89 Å), Pro480 pi-alkyl (4.42 Å)

	MRTX1133 (CID: 156124857)	-10.7	Glu105 pi-anion (3.89, 3.90 Å), Arg108 hydrogen (2.08, 2.23, 2.64 Å), Ser237 hydrogen (3.35 Å), Leu293 alkyl (4.93 Å), pi-alkyl (5.27 Å), Lys294 hydrogen (2.39 Å)
	Atogepant (CID: 72163100)	-10.7	His52 hydrogen (3.71 Å), pi-donor (2.89 Å), Asp61 pi-anion (4.61 Å), His96 hydrogen (3.77 Å), Glu105 hydrogen (3.29 Å), halogen (3.66 Å), Ala106 pi-sigma (3.57 Å), hydrogen (3.02 Å), Tyr110 hydrogen (2.70 Å), Leu293 pi-alkyl (4.65 Å), alkyl (5.40 Å), Gln329 halogen (3.06 Å), Ala340 pi-alkyl (5.48 Å), Val341 alkyl (5.35 Å), pi-sigma (3.52 Å), Arg620 hydrogen (2.01, 2.72 Å)
	Gemcitabine (CID: 60750)	-6.7	Ser449 hydrogen (3.17, 3.47 Å), Gly482 hydrogen (3.25 Å), Asp642 hydrogen (2.13 Å), pi-anion (4.75 Å), Leu712 hydrogen (1.82 Å), Asp715 hydrogen (2.76 Å), Lys718 pi-alkyl (5.20 Å)
VTI1B	Benzo[a]pyrene (CID: 2336)	-8.6	Pro29 pi-alkyl (5.37 Å), Leu32 pi-sigma (3.31, 3.74 Å), pi-alkyl (5.15, 5.29 Å), Val45 pi-alkyl (4.89, 5.18 Å), pi-sigma (3.63 Å), Phe48 pi-pi t-shaped (4.8, 5.51 Å)
	Omilancor (CID: 121299620)	-8.4	Pro29 pi-alkyl (5.50 Å), Leu32 pi-sigma (3.58, 3.64, 3.93 Å), Val45 alkyl (4.96 Å), pi-sigma (3.50 Å), Arg46 pi-alkyl (3.85 Å), pi-sigma (3.67 Å), Phe48 pi-pi t-shaped (5.10 Å), Asp49 hydrogen (2.05, 3.78 Å)
	Methylcholanthrene (CID: 1674)	-8.3	Leu32 pi-alkyl (4.54, 5.29 Å), alkyl (5.16 Å), pi-sigma (3.32, 3.44 Å), Val45 pi-alkyl (5.34 Å), alkyl (4.46, 4.81 Å), Phe48 pi-alkyl (4.10 Å), pi-pi t-shaped (4.83 Å)
	Bemcentinib (CID: 46215462)	-8.3	Leu32 alkyl (5.43 Å), pi-sigma (3.54, 3.66 Å), Leu33 pi-alkyl (5.25 Å), Val45 alkyl (4.71 Å), pi-alkyl (4.10 Å), Phe48 pi-alkyl (4.53 Å), pi-pi t-shaped (5.47 Å)
	Lifirafenib (CID: 89670174)	-8.3	Pro29 pi-alkyl (4.99 Å), Leu32 alkyl (4.52 Å), pi-alkyl (5.20 Å), pi-sigma (3.48 Å), Val45 alkyl (3.80 Å), pi-alkyl (5.25 Å), halogen (3.25 Å), Phe48 pi-pi t-shaped (4.81 Å), pi-alkyl (4.66 Å), Asp49 halogen (3.10, 3.14 Å), Glu94 pi-anion (4.87 Å)

	Gemcitabine (CID: 60750)	-5.1	Gln52 hydrogen (2.62 Å), Asn56 hydrogen (2.24 Å), Arg85 hydrogen (2.40 Å), pi-alkyl (4.24 Å), halogen (2.93, 3.62 Å), Ala89 hydrogen (3.12 Å)
YWHAQ	Bemcentinib (CID: 46215462)	-10.7	Lys49 pi-alkyl (4.91 Å), Lys120 pi-cation (3.31, 4.24 Å), Asp124 pi-anion (4.21 Å), Pro165 amide-pi stacked (4.89 Å), Ile166 amide-pi stacked (4.89 Å), Asn173 pi-donor (2.57 Å), Val176 alkyl (5.04 Å), Ile217 alkyl (4.46 Å), pi-sigma (3.67 Å), Leu220 alkyl (5.49 Å), Asn224 hydrogen (3.28 Å)
	Vanzacaftor (CID: 139399801)	-10.5	Ser45 hydrogen (2.78 Å), Arg56 pi-cation (4.37 Å), Lys49 alkyl (4.88 Å), pi-alkyl (4.65 Å), pi-cation (4.62 Å), Lys120 hydrogen (2.55 Å), Arg127 hydrogen (2.69 Å), Tyr128 hydrogen (2.94 Å), Ile166 pi-alkyl (5.40 Å), Leu172 alkyl (5.35 Å), Val176 alkyl (4.22, 4.44, 4.64 Å)
	MK-4232 C-11 (CID: 155907712)	-10.2	Asn38 pi-donor (2.76 Å), Ser45 hydrogen (2.49 Å), Lys120 pi-cation (3.70 Å), His164 hydrogen (3.06 Å), Pro165 alkyl (5.33 Å), pi-alkyl (5.20 Å), halogen (3.50 Å), Ile166 pi-alkyl (5.02 Å), halogen (3.04 Å), Gly169 hydrogen (3.54 Å), Leu172 alkyl (5.43 Å), Ile217 alkyl (4.44 Å)
	INCB13739 (CID: 66662059)	-10.0	Val46 pi-alkyl (5.46 Å), Lys49 alkyl (5.07 Å), pi-sigma (3.69 Å), Lys120 hydrogen (2.73 Å), Pro165 pi-alkyl (5.08 Å), Ile166 pi-alkyl (5.20 Å), Leu172 alkyl (5.24 Å), Ile217 pi-sigma (3.96 Å), alkyl (4.06 Å)
	RK-582 (CID: 138594194)	-10.0	Lys49 alkyl (4.42, 4.59, 5.49 Å), Tyr125 pi-alkyl (4.78 Å), Ile217 alkyl (4.34 Å)
	Gemcitabine (CID: 60750)	-6.1	Pro165 halogen (3.67 Å), Ile166 pi-alkyl (5.87 Å), Phe117 pi-pi stacked (5.16 Å), Asp213 hydrogen (2.23 Å)

Supplementary table S6. Summary of top-ranked ligands across prioritized targets, including docking score ranges, binding affinities, selected poses (pose 1), key interacting residues, interaction types, and structure/model confidence metrics. This table is provided to ensure transparency, reproducibility, and accessibility of docking results

Target	Structure/Model Confidence	Docking Score Range (kcal/mol)	Selected Ligand	Binding Affinity (kcal/mol)	Pose	Key Residues	Interaction Types
AHNAK2	1.75 Å (PDB ID: 4CN0)	-9.8 to -9.2	Conivaptan	-9.4	1	Gln179, Tyr180, Pro183, Leu175	Hydrogen bonding, hydrophobic
ANXA2	1.86 Å (PDB: 2HYU)	-10.2 to -9.4	Omilancor	-10.1	1	Arg177, Arg178, Glu180, Gly182, Pro222	π-cation, hydrogen bonding, π-alkyl
			Bemcentinib	-9.4	1	Glu112, Asp123, Lys118, Leu99	Hydrogen bonding, hydrophobic
			SYHA1813	-9.4	1	Arg167, Lys168, Val171, Glu218	π-alkyl, hydrogen bonding, π-anion
EIF2A	1.80 Å (PDB: 8DYS)	-11.5 to -10.8	Omilancor	-11.5	1	Lys126, Lys127, Arg144, Asn145, Asn147, Asn148, Val146	Hydrogen bonding, π-cation, π-alkyl
			Tapotoclax	-10.9	1	Pro85, Trp135, Lys230, Asp418	Hydrogen bonding, hydrophobic
STAM	pLDDT: 77.19 (AlphaFold: AF-Q92783-2-F1-v6)	-9.6 to -9.1	Omilancor	-9.3	1	Ile218, Ala233, Val264, Ala270, Met274	Hydrogen bonding, hydrophobic
			Bemcentinib	-9.6	1	Ile218, Asp267, Ala270, Met274	Hydrogen bonding, π-sigma, hydrophobic
			APTO-253	-9.2	1	Arg216, Ile218, Tyr219, Ala233, Pro272, Met274	π–π interactions, hydrophobic
