# Supplementary material for: Knowledge Discovery and Drug-Repurposing Framework for Pancreatic Ductal Adenocarcinoma: Molecular Networking and Computational Docking
Source: Comput Struct Biotechnol J. 2026 May 5;35(1):0067. doi: 10.34133/csbj.0067 (PMC13139727; doi:10.34133/csbj.0067)
Supplement: Supplementary 1 — Figs. S1 to S28 Tables S1 to S6 [file csbj.0067.f1.zip › Supplementary Figure Captions.pdf]

Supplementary Figure 1. Structural visualization of the binding interactions between ACADSB and selected candidate compounds, alongside the positive control gemcitabine. Panels represent: A) Hypericin, B) Conivaptan, C) Adavivint, D) Lumacaftor, E) LUT-014, and F) Gemcitabine. Dashed interaction lines are color-coded to indicate the type of bonds: green – hydrogen bonds, purple/pink – hydrophobic interactions, orange – electrostatic interactions, blue – halogen bonds. This convention is maintained across all supplementary figure captions.

Supplementary Figure 2. Structural visualization of the binding interactions between the ACTR1A and selected candidate compounds, alongside the positive control gemcitabine. Panels represent: A) Bifeprunox, B) Canagliflozin, C) Hypericin, D) Vebreltinib, E) Cirtuvivint, and F) Gemcitabine.

Supplementary Figure 3. Structural visualization of the binding interactions between ANXA3 and selected candidate compounds, alongside the positive control gemcitabine. Panels represent: A) HRS-4642, B) MRTX1133, C) AZD-5991, D) Danicopan, E) Hypericin, and F) Gemcitabine.

Supplementary Figure 4. Structural visualization of the binding interactions between ATP6V1F and selected candidate compounds, alongside the positive control gemcitabine. Panels represent: A) Atogepant, B) AJ-030/12105289, C) 3-[[4-imidazol-1-yl-6-(4-oxidanylpiperidin-1-yl)-1,3,5-triazin-2-yl]amino]-4-methyl-~{N}-[3-(trifluoromethyl)phenyl]benzamide, D) GSK-1521498, E) Nilotinib, and F) Gemcitabine.

Supplementary Figure 5. Structural visualization of the binding interactions between GSN and selected candidate compounds, alongside the positive control gemcitabine. Panels represent: A) Dihydroergotamine, B) Vevorisertib, C) Cirtuvivint, D) Lifirafenib, E) MK-3207, and F) Gemcitabine.

Supplementary Figure 6. Structural visualization of the binding interactions between HPCAL1 and selected candidate compounds, alongside the positive control gemcitabine. Panels represent: A) Milbemycin A3, B) Enzastaurin, C) Tirilazad, D) BMS-955176, E) MK-1084, and F) Gemcitabine.

Supplementary Figure 7. Structural visualization of the binding interactions between MVB12A and selected candidate compounds, alongside the positive control gemcitabine. Panels represent: A) Hypericin, B) Apto-253, C) Tirilazad, D) Cepharanthine, E) Lestaurtinib, and F) Gemcitabine.

Supplementary Figure 8. Structural visualization of the binding interactions between MYH14 and selected candidate compounds, alongside the positive control gemcitabine. Panels represent: A) Casopitant, B) GLPG-1205, C) HRS-4642, D) Enoxolone, E) Bemcentinib, and F) Gemcitabine.

Supplementary Figure 9. Structural visualization of the binding interactions between MYO1C and selected candidate compounds, alongside the positive control gemcitabine. Panels represent: A) MK-4232 C-11, B) Brigimadlin, C) Conivaptan, D) Atogepant, E) Vanzacaftor, and F) Gemcitabine.

Supplementary Figure 10. Structural visualization of the binding interactions between OTUB1 and selected candidate compounds, alongside the positive control gemcitabine. Panels

represent: A) Bemcentinib, B) 1144072-40-1, C) 15-PGDH-IN-1, D) Tapotoclax, E) Ubrogepant, and F) Gemcitabine.

Supplementary Figure 11. Structural visualization of the binding interactions between PACSIN2 and selected candidate compounds, alongside the positive control gemcitabine. Panels represent: A) HRS-4642, B) MRTX1133, C) 2247950-42-9, D) Zavegepant, E) Nilotinib, and F) Gemcitabine.

Supplementary Figure 12. Structural visualization of the binding interactions between PCCA and selected candidate compounds, alongside the positive control gemcitabine. Panels represent: A) 1144072-40-1, B) Methylcholanthrene, C) Boditrectinib, D) PF-07284892, E) Tirilazad, and F) Gemcitabine.

Supplementary Figure 13. Structural visualization of the binding interactions between PCK2 and selected candidate compounds, alongside the positive control gemcitabine. Panels represent: A) HRS-4642, B) Bosmolisib, C) Dihydroergotamine, D) Benzo[a]pyrene, E) Galicaftor, and F) Gemcitabine.

Supplementary Figure 14. Structural visualization of the binding interactions between PDHB and selected candidate compounds, alongside the positive control gemcitabine. Panels represent: A) Lifirafenib, B) Milbemycin A3, C) Dihydroergotamine, D) Natamycin, E) Padnarsertib, and F) Gemcitabine.

Supplementary Figure 15. Structural visualization of the binding interactions between PLP2 and selected candidate compounds, alongside the positive control gemcitabine. Panels represent: A) Tirilazad, B) Bemcentinib, C) MK-4232 C-11, D) GRT6010, E) HRS-4642, and F) Gemcitabine.

Supplementary Figure 16. Structural visualization of the binding interactions between POLR2H and selected candidate compounds, alongside the positive control gemcitabine. Panels represent: A) Lestaurtinib, B) UCN-01, C) TMC-647055, D) Zavegepant, E) Tivantinib, and F) Gemcitabine.

Supplementary Figure 17. Structural visualization of the binding interactions between SCO1 and selected candidate compounds, alongside the positive control gemcitabine. Panels represent: A) Tirilazad, B) Tapotoclax, C) Padnarsertib, D) Quarfloxin, E) Atogepant, and F) Gemcitabine.

Supplementary Figure 18. Structural visualization of the binding interactions between SCPEP1 and selected candidate compounds, alongside the positive control gemcitabine. Panels represent: A) Omilancor, B) Zavegepant, C) Atogepant, D) ABN401, E) ITI-214, and F) Gemcitabine.

Supplementary Figure 19. Structural visualization of the binding interactions between SERPINB6 and selected candidate compounds, alongside the positive control gemcitabine. Panels represent: A) Quarfloxin, B) BMS-986142, C) Hypericin, D) RK-582, E) 3-[[4-imidazol-1-yl-6-(4-oxidanylpiperidin-1-yl)-1,3,5-triazin-2-yl]amino]-4-methyl-~{N}-[3-(trifluoromethyl)phenyl]benzamide, and F) Gemcitabine.

Supplementary Figure 20. Structural visualization of the binding interactions between SFXN2 and selected candidate compounds, alongside the positive control gemcitabine. Panels represent: A) Padnarsertib, B) Zavegepant, C) Laniquidar, D) SYHA1813, E) JNJ-49095397 (RV568), and F) Gemcitabine.

Supplementary Figure 21. Structural visualization of the binding interactions between SH3BGRL3 and selected candidate compounds, alongside the positive control gemcitabine. Panels represent: A) Rebastinib, B) Padnarsertib, C) Bemcentinib, D) Zongertinib, E) RAF-265, and F) Gemcitabine.

Supplementary Figure 22. Structural visualization of the binding interactions between SNAP23 and selected candidate compounds, alongside the positive control gemcitabine. Panels represent: A) Nilotinib, B) RAF-265, C) 3-[[4-imidazol-1-yl-6-(4-oxidanylpiperidin-1-yl)-1,3,5-triazin-2-yl]amino]-4-methyl-~{N}-[3-(trifluoromethyl)phenyl]benzamide, D) MK-3207, E) Bemcentinib, and F) Gemcitabine.

Supplementary Figure 23. Structural visualization of the binding interactions between SPTAN1 and selected candidate compounds, alongside the positive control gemcitabine. Panels represent: A) Lomitapide, B) Adavivint, C) ONO-7475, D) Omilancor, E) Relacorilant, and F) Gemcitabine.

Supplementary Figure 24. Structural visualization of the binding interactions between SURF1 and selected candidate compounds, alongside the positive control gemcitabine. Panels represent: A) Bemcentinib, B) HRS-4642, C) MK-3207, D) Telcagepant, E) Dihydroergotamine, and F) Gemcitabine.

Supplementary Figure 25. Structural visualization of the binding interactions between TIMM50 and selected candidate compounds, alongside the positive control gemcitabine. Panels represent: A) Cirtuvivint, B) Omilancor, C) MK-3207, D) Risvodetinib, E) Bemcentinib, and F) Gemcitabine.

Supplementary Figure 26. Structural visualization of the binding interactions between VIL1 and selected candidate compounds, alongside the positive control gemcitabine. Panels represent: A) Bemcentinib, B) Padnarsertib, C) Tirilazad, D) MRTX1133, E) Atogepant, and F) Gemcitabine.

Supplementary Figure 27. Structural visualization of the binding interactions between VTI1B and selected candidate compounds, alongside the positive control gemcitabine. Panels represent: A) Benzo[a]pyrene, B) Omilancor, C) Methylcholanthrene, D) Bemcentinib, E) Lifirafenib, and F) Gemcitabine.

Supplementary Figure 28. Structural visualization of the binding interactions between YWHAQ and selected candidate compounds, alongside the positive control gemcitabine. Panels represent: A) Bemcentinib, B) Vanzacaftor, C) MK-4232 C-11, D) INCB13739, E) RK-582, and F) Gemcitabine.
